# Physical Activity interventions on health-related quality of life (HR-QoL) in major depressive disorder (MDD): a systematic review and meta-analysis of randomized controlled trials

**DOI:** 10.3389/fpsyt.2026.1806489

**Published:** 2026-05-22

**Authors:** Jing Peng, Jing Tong Huang, Chunbi Hu, Shiling Zhu, Hongyan Huang, Huixia Liao, Qiong Zheng, Bo Yang, Qin Liu

**Affiliations:** 1 Department of Geriatrics, Chongqing Mental Health Center, Chongqing, China; 2 Department of Rehabilitation Medicine, Chongqing Mental Health Center, Chongqing, China; 3 Department of Nursing, Chongqing Mental Health Center, Chongqing, China; 4 Sleep Medical Center, Chongqing Mental Health Center, Chongqing, China

**Keywords:** Exercise, health related quality of life, major depressive disorder, physical activity, psychiatry

## Abstract

**Background:**

Major Depressive Disorder (MDD) causes global disability and economic burden. Standard treatments often yield incomplete recovery with impaired health-related quality of life (HR-QoL). This meta-analysis evaluates exercise interventions’ effects on HR-QoL in adults with clinically diagnosed MDD.

**Methods:**

Following PRISMA 2020 guidelines, randomized controlled trials (RCTs) of adults (≥18 years) with MDD were included if they evaluated physical activity/exercise interventions (e.g., aerobic, resistance, yoga, Tai Chi) versus active or non-active controls with HR-QoL outcomes. Comprehensively the main four databases and grey literature searches were conducted. Two reviewers independently screened studies, extracted data, and assessed risk of bias using the Cochrane RoB-2 tool. Statistical analysis performed by STAT-17.

**Results:**

Finally, 11 RCTs were included (mean age about 48.9 years; majority female participants (68%)). The meta-analysis showed that exercise interventions significantly improved overall HR-QoL immediately after treatment (SMD = 0.31 [0.16, 0.46], I² = 0.00%). At follow-up, exercise was also associated with a significant improvement in HR-QoL (SMD = 0.41 [0.13, 0.70], I² = 34.42%). Domain-level analyses indicated significant improvements favoring exercise in overall (SMD = 0.25 [0.05, 0.45], I² = 0%), physical (SMD = 0.50 [0.10, 0.91], I² = 48.18%), psychological (SMD = 0.58 [0.24, 0.92], I² = 0%), and emotional (SMD = 0.51 [0.11, 0.91], I² = 21.03%) domains, while the social domain showed no significant difference (SMD = 0.11 [–0.34, 0.57], I² = 0%).

**Conclusion:**

Exercise interventions, primarily structured and supervised, may improve short-term HR-QoL in adults with major depressive disorder, particularly in the physical and psychological domains. However, long-term effects remain uncertain, and current evidence does not support the superiority of any specific exercise modality. Given the limited number of studies, heterogeneity in control conditions, and variability in HR-QoL measures, further high-quality randomized controlled trials with longer follow-up are needed.

## 1 Introduction

Major Depressive Disorder (MDD) is a frequent, serious psychiatric disorder, characterized by a low mood, anhedonia, cognitive dysfunction, and impaired functioning (1). As reported by the World Health Organization, when last reported in 2019, it was estimated that approximately 280 million people were living with depression annually, which is equivalent to roughly 5% of the adult population globally (2). Additionally, based on population studies, it is likely that unipolar major depression has a lifetime prevalence of around 15-20%, especially taking into account earlier onset in adolescence and young adulthood (3). In fact, depressive disorders were reported to be among the leading causes of years lived with disability (YLDs) worldwide in 2021 (4). Economically, MDD incurs very large costs associated with increased healthcare use and large losses in the loss of workplace productivity, with national estimates in high income countries being in the tens or hundreds of billions of dollars per year (5).

More importantly, health related quality of life (HR-QoL) in persons with MDD is substantially and consistently lower in physical, psychological, social, and occupational domains compared to the general population (6). Physiological factors will generally include areas like pain, fatigue, sleep, and ability to function physically; psychological factors will include areas such as mood, thinking skills, self-respect, and emotional health; social factors will address interactions with other people and involvement socially; and occupational factors will address the ability to work effectively (7). Despite the patients recovering from their symptoms, many still complain about residual depressive symptoms like persistently feeling sad, sleep problems, tiredness, anxiousness, poor concentration, and inability to function well, thus affecting HR-QoL (8). Since HR-QoL not only represents the “count of symptoms” but also represents a more holistic view of recovery, it is increasingly viewed as one of the most important outcomes in clinical trials for MDD and health services research (9).

Standard treatments for MDD include pharmacotherapy with selective serotonin reuptake inhibitors (SSRIs) or serotonin–norepinephrine reuptake inhibitors (SNRIs), psychotherapies such as cognitive behavioral therapy (CBT) and interpersonal therapy (IPT), and newer combined approaches (10). Although antidepressants reduce depressive symptoms in many patients, they commonly have a delayed onset of action and a substantial minority of patients exhibit partial response or nonresponse (11). Not to mention that besides of the therapeutic effects, these interventions make several side effects like digestive and appetite changes, sleep disturbances, sexual dysfunction, emotional blunting and etc. that sometimes handling these issues are problematic (12). Additionally, the relapse rates after discontinuation remain appreciable and long-term adherence is often undermined by adverse effects such as sexual dysfunction, weight gain, sleep disturbance, and emotional blunting (13). However, psychotherapies are another effective approach for many MDD patients but are limited in reach by therapist availability, due to its treatment cost, waiting lists, and patient time burden (14). On the other hand, habitual physical activity and structured exercise improve a wide variety of cardiometabolic, musculoskeletal, and some other chronic diseases (15–17). At the neurobiological level, exercise increases neurotrophic factors including brain-derived neurotrophic factor (BDNF), modulates monoamine systems, reduces systemic inflammation, and promotes neuroplasticity, all of which are part of the pathophysiology of depression. In addition, exercise has been shown to reduce core depressive symptoms, improve sleep quality, and attenuate inflammatory processes, which are closely linked to MDD symptomatology and overall disease burden (18). Randomized trials and meta-analyses have consistently demonstrated that exercise interventions (aerobic and resistance training) result in clinically meaningful reductions in depressive symptom severity relative to inactive control groups (19–21). Interestingly, some studies focused on MDD samples report HR-QoL gains alongside symptom improvement after supervised aerobic or resistance programs, suggesting exercise may promote broader recovery beyond symptom reduction (22, 23).

Despite consistent evidence for symptom reduction, relatively few meta-analyses have focused explicitly on HR-QoL outcomes in trials restricted to patients meeting formal MDD diagnostic criteria (24–26). For example, Pérez Bedoya et al. (2023) explored the effects of exercise on HR-QoL but included only two studies (27). Moreover, many trials report HR-QoL as a secondary outcome, limiting the ability to draw firm conclusions regarding its magnitude and sustainability (28). Accordingly, this study aims to systematically evaluate whether exercise interventions improve HR-QoL and its domains in adults with diagnosed MDD, while also assessing the quality and consistency of the available evidence.

## 2 Methods

### 2.1 Protocol and reporting

We followed PRISMA 2020 guidelines for systematic reviews and meta-analyses (29).

### 2.2 Eligibility criteria (PICOS)

- Population. Adults (≥18 years) with unipolar major depressive disorder (MDD) diagnosed by standardized criteria (DSM/ICD) or validated clinical interview (e.g., SCID, MINI). Studies including bipolar depression or mixed psychiatric populations were excluded.
- Interventions. Interventions involving any type of structured and repetitive physical activity, which can be defined as exercise whether as monotherapy or an adjuvant to other types of care, including aerobic and strength training exercises, combined exercises, and mind-body activities such as yoga, tai chi, qigong, and laughter yoga. According to previous definitions, exercise was considered one form of physical activity characterized by being structured and organized. Mind-body exercises were thus included as structured exercises because of their standardized form and clinical settings. Psychotherapeutic and mindfulness therapies lacking a body movement element were not included in this review.
- Comparators. Active comparators (e.g., health education, attention control) or non-active controls (e.g., treatment-as-usual [TAU], waitlist, no intervention). Trials comparing two active exercise interventions without a non-exercise comparator were excluded from quantitative synthesis.
- Outcomes. HR-QoL measured with validated instruments (e.g., SF-36/12/20, WHOQOL-BREF, Q-LES-Q-SF, WHO-DAS II), which capture multiple domains including physical functioning (e.g., mobility, pain, energy), psychological well-being (e.g., mood, cognition), and social functioning. While depressive symptom scales (e.g., HAM-D, HDRS, PHQ-9) and physical measures were reported in several studies, these were treated as secondary outcomes and not quantitatively synthesized in this meta-analysis. Trials with no HR-QoL data were included only in narrative mapping and sensitivity counts.
- Study design. RCTs (parallel-group). Cluster and crossover RCTs were eligible if data allowed unit-of-analysis correction. Non-randomized studies were excluded from quantitative synthesis but could be summarized narratively if relevant.
- Setting & language. Any setting (inpatient/outpatient/community). Just English language, without date restrictions.

### 2.3 Information sources and search strategy

We conducted a comprehensive literature search across major electronic databases including PubMed, Scopus, Web of Science, Cochrane Central, and Psychinfo from inception to September 18, 2025. The search strategy combined terms related to depression (e.g., “major depressive disorder,” “depressive symptoms”), quality of life (e.g., “health-related quality of life,” “HR-QoL “), exercise/physical activity (e.g., “exercise,” “yoga,” “tai chi,” “qigong,” “physical activity”), and randomized controlled trials (e.g., “random* assigned,” “double-blind,” “control”). Database-specific subject headings (e.g., MeSH, Emtree) and keyword variations were used where appropriate. The full search strings are provided in **Supplementary Material**.

To supplement the database search, we conducted a grey literature search through ClinicalTrials.gov, and performed manual searches in Google Scholar. We also screened the reference lists of all included studies and of relevant systematic reviews (25, 27, 30–35) to identify additional eligible trials. All records were managed using reference management software, with duplicates removed prior to screening.

### 2.4 Study selection

All records retrieved from the database and grey literature searches were imported into EndNote 21 for reference management, where duplicates were identified and removed. The remaining unique records were then uploaded into Rayyan (36), a web-based tool for systematic reviews, to facilitate screening. Screening was performed in two stages. In the first stage, titles and abstracts were independently reviewed by two investigators to exclude clearly irrelevant articles. In the second stage, the full texts of potentially eligible studies were obtained and assessed in detail against the prespecified PICOS criteria. Disagreements at both stages were resolved through discussion or by consultation with a third reviewer. Reasons for exclusion at the full-text stage were documented, and the overall selection process was summarized in a PRISMA flow diagram.

### 2.5 Data extraction

Two reviewers independently extracted data using a piloted form. Items included: bibliographic details; country and setting; design; sample size; age and sex; MDD diagnostic method and severity; comorbidities; intervention type, dose (minutes/session, sessions/week), duration, supervision, delivery mode (group/individual); comparator type (active vs non-active); adjunct vs monotherapy; HR-QoL instrument(s) and domain(s); follow-up timings; Perceived Quality; Potential Mediators, HR-QoL outcomes as primary outcome, and other psychiatric outcomes as secondary outcomes were reported in the **Table 1**. Statistical data required for meta-analysis (means, SD/SE, CIs, change scores, event counts); analysis population (ITT/PP); and funding. When multiple reports described the same trial, we collated data to avoid duplication. Authors were contacted for missing outcome statistics when needed.

**Table 1:** Details of the included studies.

Study	Follow-up timings	Perceived quality	Potential mediators	Primary outcome	Secondary outcomes
Vollbehr et al., 2022 (43)	Baseline, post-intervention about 10–15 weeks, 6 months, 12 months	High Satisfaction; Strong fidelity	Rumination, Mindfulness, self-compassion	No group significant difference; significant improvement over time	Improved psychological function
Lavretsky et al., 2022 (40)	Baseline, 3 months, 6 months	High adherence (about 80 %)	Resilience, baseline severity, sex	The HR-QoL in both groups significantly improved; with no between-group significant difference	Depressive symptoms remission through ≤6: 3 months with no significant different
Bressington et al., 2019 (38)	Baseline, 4 weeks, 3-month	High satisfaction:	Stress reduction, social connection improvement	Improved trend toward better self-reported physical and mental health at 4-weeks and 3-month follow-up, but not significant compared with TAU	Depression and anxiety remission (within-group)
Haller et al., 2018 (45)	6–12 days, and 8 weeks	Feasible, with high adherence	Engagement, training dose	significant improvement in Emotional and social functioning, no significant improvement in total score	Depression improved in the intervention group with no significant difference vs controls
Uebelacker et al., 2017 (46)	3 & 6 months	Higley fidelity, good engagement	Practice, dose, adherence	Improved overall HR-QoL across non-baseline timepoints	lower depressive symptoms & improved social & role-functioning compared with HLW
Schuch et al., 2015 (41)	Second week, Discharge	NR	NR	significant improvement in Physical and psychological domains in the exercise group vs TAU; not significantly different in social relationships & environment domains	Exercise group had greater reduction in depressive symptoms at second week and discharge
Kinser et al., 2014 (44)	Weeks 2, 4, 6, 8 (depression); Weeks 0, 4, 8 (stress, anxiety, HR-QoL); 52 weeks (1 year)	High Acceptability	Sustained practice	HR-QoL showed improvement over time in both groups; in long-term suggested better mental HR-QoL at 52 weeks	Depression severity and rumination decreased more in yoga group at 52 weeks; stress, anxiety improved in both groups
Yeung et al., 2012 (42)	Week 6, Week 12	Good Adherence (73%)	NR	HR-QoL scores improved from baseline in Tai Chi group but not significantly different from control at week 12	no significant between-group differences in depressive symptoms
Mota-Pereira et al., 2011 (47)	Baseline (T0) and 12 weeks (T1; final observation)	High adherence (91%); good acceptance and engagement	Remission Status	Exercise group improved social domain & physical functioning vs controls	Exercise group showed significantly remission in depression and anxiety
Lavretsky et al., 2011 (39)	Every 2 weeks, post-intervention at 10 weeks	High credible and satisfying; adherence high, dropout low (7%)	stress regulation	Significant improvement in physical functioning for TCC vs HE	TCC+SSRI showed greater reductions in depression severity
Carta et al., 2008 (23)	2, 4, 6, and 8 months	High adherence (71%), and good acceptability	NR	Significant improvement in the Physical Health domain in the PA group	Improvements in depression severity, global functioning, in PA group vs control

PA, Physical Activity; TCC, Tai Chi Chih; NB, No Blinding; HE, Health Education; SB, Single Blind; LY, Laughter Yoga; HEW, Health Education and Wellness; MC, Multi Centre; MYI, Mindfulness-Based Yoga Intervention; HR-QoL, Health-Related Quality of Life; HLW, Health and Lifestyle Workshop; CSQ-8, Client Satisfaction Questionnaire–8; TAU, Treatment as Usual; NR, Not Reported; SSRI, Selective Serotonin Reuptake Inhibitor; SNRI, Serotonin–Norepinephrine Reuptake Inhibitor; QoL, Quality of Life; MDD, Major Depressive Disorder.

### 2.6 Risk of bias assessment

Risk of bias was assessed independently by two reviewers using the Cochrane Risk of Bias 2 (RoB 2) tool, covering five domains: randomization process, deviations from intended interventions, missing outcome data, outcome measurement, and selection of the reported result. Each domain was rated as low risk, some concerns, or high risk, with overall judgments derived using the RoB 2 algorithm (37). Disagreements were resolved by consensus or a third reviewer. Results were displayed with traffic-light plots for individual studies and a summary plot for domain-level judgments, providing a clear overview of methodological quality.

### 2.7 Certainty of evidence assessment

The certainty of evidence for the main outcomes was assessed using the GRADE approach and summarized in a Summary of Findings table (**Table 2**). In accordance with Cochrane guidance, certainty was evaluated separately for each outcome across five domains: risk of bias, inconsistency, indirectness, imprecision, and publication bias. Randomized controlled trials were initially considered high-certainty evidence and were downgraded when concerns were identified in one or more of these domains. Certainty ratings were categorized as high, moderate, low, or very low. The main outcomes assessed in **Table 2** were HR-QoL total immediately post-intervention, overall HR-QoL at the last follow-up, and the domain-specific outcomes of overall/global, physical, psychological, social, and emotional HR-QoL.

**Table 2:** GRADE Summary of Findings for the effects of exercise interventions on health-related quality of life (HR-QoL) in adults with major depressive disorder.

Outcome	N of studies	Participants	Effect estimate (SMD, 95% CI)	Certainty of evidence (GRADE)
HR-QoL total	8	637	0.31 [0.16, 0.46]	Moderate ⊕⊕⊕◯
HR-QoL total, last follow-up	4	408	0.41 [0.13, 0.70]	Moderate ⊕⊕⊕◯
Overall HR-QoL domain	3	388	0.25 [0.05, 0.45]	Moderate ⊕⊕⊕◯
Physical HR-QoL domain	4	193	0.50 [0.10, 0.91]	Low ⊕⊕◯◯
Psychological HR-QoL domain	4	138	0.58 [0.24, 0.92]	Moderate ⊕⊕⊕◯
Social HR-QoL domain	2	70	0.11 [−0.34, 0.57]	Low ⊕⊕◯◯
Emotional HR-QoL domain	2	123	0.51 [0.11, 0.91]	Low ⊕⊕◯◯

a. Risk of bias: downgraded one level for all outcomes because all included studies had some concerns in the RoB 2 assessment, particularly in the measurement of outcomes domain due to reliance on self-reported HR-QoL and lack of blinding.

b. Inconsistency: not downgraded for the main outcomes (post-intervention and follow-up HR-QoL), as heterogeneity was low to moderate (I² = 0% and 34.42%, respectively). Domain-level analyses also showed no substantial inconsistency.

c. Imprecision: downgraded for physical, social, and emotional domains due to small sample sizes, limited number of studies, and/or wide confidence intervals. The social domain was further downgraded because the confidence interval included the null effect.

d. Indirectness: not downgraded, as the included studies directly addressed the target population (adults with MDD), intervention (exercise-based), comparator, and outcome (HR-QoL).

e. Publication bias: not downgraded, as funnel plots were symmetric and Egger’s and Begg’s tests did not indicate significant publication bias for the main outcomes.

### 2.8 Statistical analysis

We synthesized continuous HR-QoL outcomes using standardized mean differences (Hedges g) with 95% CIs, coded so that positive values favored exercise. When both change-from-baseline and final values were available, we prioritized change scores; otherwise, we used final values and converted SEs/CIs to SDs with standard formulas. The primary analyses estimated pooled effects at two time points: immediately post-intervention (8 studies contributing 16 effect sizes) and at the last available follow-up (4 studies contributing 5 effect sizes). We fit random-effects models with REML estimation and inverse-variance weighting; between-study variance was expressed heterogeneity as I² (with Cochran’s Q reported). Where at least two effect sizes were available, we conducted domain-specific meta-analyses (overall/global, physical, psychological, emotional, social). Sensitivity analyses included leave-one-out (iteratively removing each study) and re-fitting the primary models. Small-study/publication bias was examined visually with funnel plots and formally using Egger’s regression and Begg’s rank tests for all meta-analyses where sufficient studies were available, and, when asymmetry suggested potential bias, we applied Duval and Tweedie’s trim-and-fill as a corrective sensitivity analysis. We performed *a priori* subgroup meta-analyses by: Region (America, Europe, Asia), Age (<60 vs ≥60 years), Sex (female-only vs both sexes), Setting (inpatient, outpatient, mixed), Intervention type (yoga; tai chi/qigong; aerobic/physical exercise), Session time (≤1 hour vs >1 hour), Frequency per week (≤2 vs >2 sessions), Health-education co-intervention (yes vs no), Program duration (<12 vs ≥12 weeks), and HR-QoL instrument (SF-12, SF-36, WHOQOL, Q-LES-Q-SF). Between-subgroup differences were assessed using the Qb test within a mixed-effects framework. All tests were two-sided with α = 0.05. Analyses were conducted in Stata/SE 17.

## 3 Results

### 3.1 Study selection

**Figure 1:**
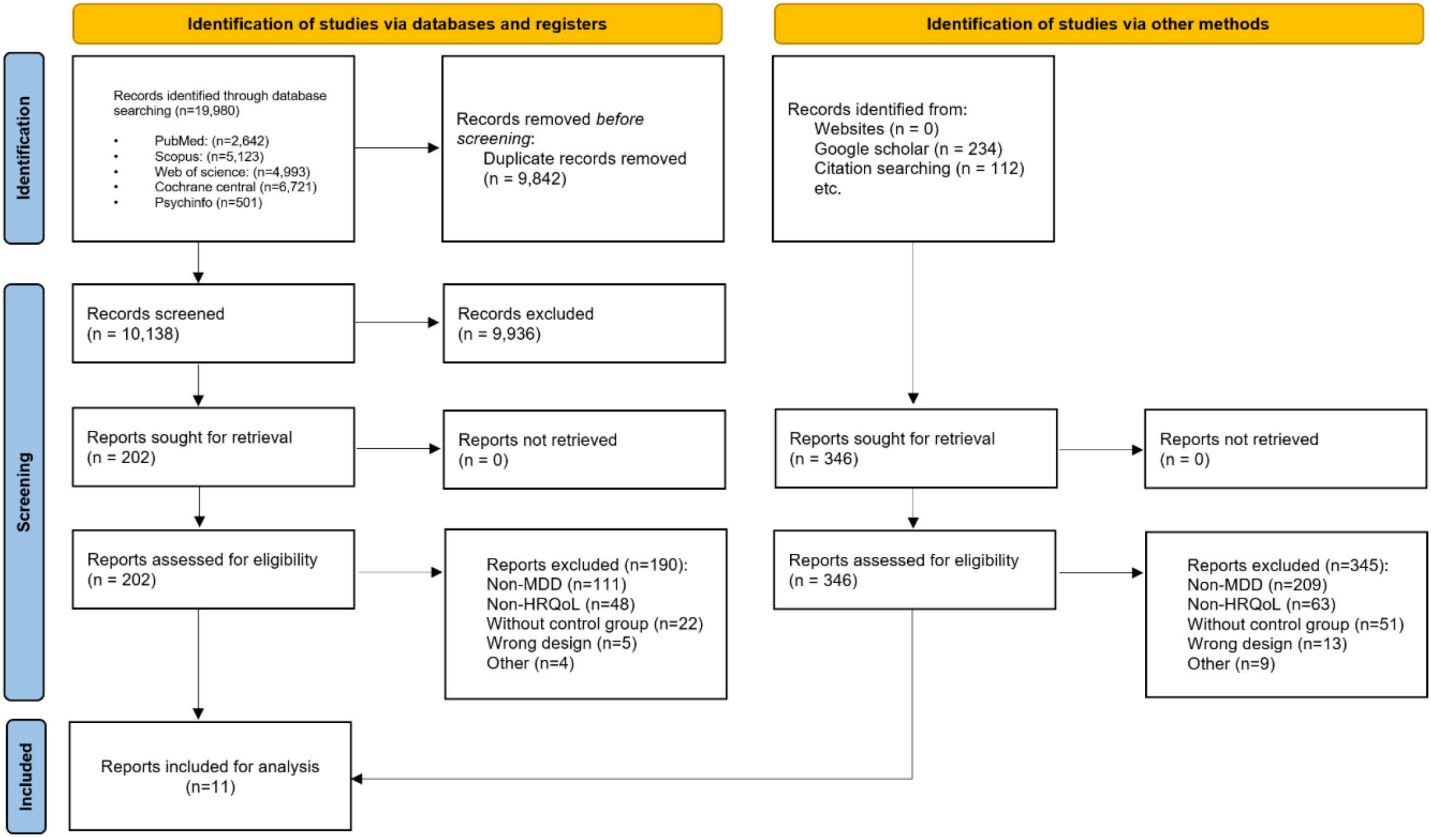
PRISMA diagram of all included studies.

The study selection process is presented in the PRISMA flow diagram (**Figure 1**). A total of 19,098 records were initially identified through database searches, of which 9,842 duplicates were removed, leaving 10,138 records for screening. After title and abstract screening, 9,936 records were excluded, and 202 full-text articles were assessed for eligibility. Of these, 190 were excluded due to reasons such as non-MDD populations (n=111), no HR-QoL outcomes (n=48), absence of a control group (n=22), wrong study design (n=5), or other reasons (n=4). In parallel, 346 additional records were identified through other sources (websites, Google Scholar, citation searching) and assessed for eligibility, of which 345 were excluded for similar reasons [non-MDD (n=200), no HR-QoL outcomes (n=53), absence of a control group (n=51), wrong study design (n=13), or other reasons (n=9)]. Ultimately, 11 studies (23, 38–47) met the inclusion criteria and were included in the systematic review and meta-analysis.

### 3.2 Study characteristics

11 RCTs were included (23, 38–47), all conducted between 2008 and 2022, in six countries: five from the USA (39, 40, 42, 44, 46), one from Netherlands (43), one from Germany (45), one from Brazil (41), one from Hong Kong (38), one from Portugal (47), and one from Italy (23). All studies used parallel-group randomized designs, with the majority being single-centre (23, 38, 39, 41, 45–47), and two multi-centres (40, 43). The blinding method was different through different studies, with five studies using double-blind (39–41, 45, 46), two studies applied single-blinding method (42, 46), however several studies were non-blinded (23, 38, 44, 47). Most studies were conducted in outpatient or community settings, including three studies using hospital-based outpatient clinics (39, 41, 46), two studies from university or clinical research units (23, 47), two studies included urban community centres (42, 44), the other two studies included hospital inpatients (40, 41).

Sample sizes ranged from 10 to 178 participants, overall, 637 patients were included in the systematic review. The mean ages ranged from 25.05 ± 4.64 years in Vollbehr et al., 2022 (43) study to 70.57 ± 7.34 years from Lavretsky et al., 2011 (39) study, however, mota-pereira et al., 2011 and carta et al., 2008 didn’t report their mean ± SDs in the full text (23, 47). The overall mean ± SD of age across all of the 11 included studies was 48.9 ± 10.4. The majority of participants were female, with some samples being entirely female (23, 43, 44) and others mixed-gender (23, 38–42, 45–47).

The depression severity of included patients at baseline was measured by standardized instruments, such as: four studies used HAM-D (23, 40, 41, 47), two studies HDRS (39, 43), two studies QIDS (45, 46), one study PHQ-9 (44), and DASS-21 (38).

The comorbidities of included patients were varied across the included studies. Some studies excluded all comorbidities (43, 44, 46), while others permitted specific comorbidities, such as generalized anxiety disorder (GAD), social phobia (SP), and panic disorder (PD) (23) or physical comorbidities (hypertension, diabetes) (38).

Interventions that were applied in the included studies were somehow with exercise-bass. One study intervened mindfulness-based Yoga Interventions (MYI) (43), three studies Tai Chi Chih (TCC) (39, 40, 42), one study Laughter Yoga (LY) (38), one study Hatha Yoga (46), two studies aerobic exercise (41, 47), and two studies structured physical activity programs (23, 45). On the other hand, the duration of interventions ranged from 4 weeks (Bressington et al., 2019 (38)) to 32 weeks (Carta et al., 2008 (23)), with session frequencies varying from one to five times per week.

Qualified professionals delivered all interventions across all include studies. Three studies employed certified Tai Chi Chih trainers (39), two studies employed registered yoga teachers (44, 46), one study employed psychologists and yoga teachers (43), one study employed sports therapists (45), one study employed trained physiologists (41), and one another study employed skilled instructors supervised by physicians and psychologists (23).

Three studies used treatment-as-usual (TAU) (41, 43, 45), three studies used health education/wellness (HEW) (39, 40, 42), one study used waitlist control (42), two studies used pharmacotherapy-only (23, 47), and two studies used usual care (38, 44), as their comparator. Most interventions were group-based, promoting social engagement (23, 38–40, 42, 43, 46), whereas others were individualized (41, 45, 47).

Follow-up durations across included studies, ranged from 8 weeks to 12 months, enabling evaluation of both short- and long-term effects. Most studies assessed outcomes at baseline and post-intervention, with several including extended follow-ups at 3, 6, or 12 months (40, 43, 44, 46). Although, Vollbehr et al., 2022 (43) evaluated outcomes at baseline, post-intervention (10–15 weeks), 6 and 12 months, while Kinser et al., 2014 included assessments up to 52 weeks (44). Conversely, shorter-term studies such as Bressington et al., 2019 assessed participants at 4 weeks and 3 months (38), and Haller et al., 2018 at 6–12 days and 8 weeks (**Table 3**) (45).

**Table 3:** General and clinical characteristics of all included studies.

Study	Country	Design	Setting	Population			Comorbidities	Intervention		Instruction	MDD Severity (scale, degree)	Comparison		
				Sample Size	Age (mean ± SD)	Sex (F/M)		Type	Dose & Duration			Definition	Delivery mode	Comparator type
Vollbehr et al., 2022 (43)	Netherlands	R, P TAU-C, MC	Outpatient	171	25.05 ± 4.64	171/0	Non, psychiatric were exclude	MYI+ TAU	9 × 1.5 hr; home practice 30–45 min/day	Psychologist and Yoga Teacher	HDRS, 18.63 ± 5.94	TAU only	Group	Active + non-active
Lavretsky et al., 2022 (40)	USA	RCT, SC, P, DB, AC	Hospital & community	178	69.3 ± 6.6	129/ 49	Non, psychiatric were exclude	TCC	60 min/week + 20 min/day home practice, 12-weeks	Certified Tai Chi Chih trainers	HAM-D, 19.1 ± 3.9	HEW	Group (6–8 participants)	Active
Bressington et al., 2019 (38)	Hong Kong	RCT, SC, P, NB,	Hospital	50	47.96 ± 10.99	35/15	physical comorbidities (mostly HTN, DM)	LY	45 min/ × 2 /week × 4 weeks (8 sessions total); daily home practice 4 weeks	1 certified lead LY trainer + 3 co-investigators	DASS-21, 25.39 ± 2.68	TAU	Group (max 12 participants)	Active usual care
Haller et al., 2018 (45)	Germany	RCT, SC, P, DB, AC	outpatient clinic	20	45 ± 8	13/7	NR	Web-based exercise	Endurance: 30–60 min/session, 2–3x/week; Strength: 2x/week; 8 weeks	Sports therapist	QIDS-SR 16 ± 3; QIDS-C 14 ± 3	TAU	Group	active
Uebelacker et al., 2017 (46)	USA	RCT, SC, P, SB, AC	hospital outpatients	122	44.49 ± 12.95	84/38	NR, psychiatric were excluded	Hatha Yoga	80 min/session × 1–2 sessions/week × 10 weeks; encouraged home practice 10-weeks	Registered Yoga Teachers	QIDS, overall, in 8–17 range	HLW	Goup	Active
Schuch et al., 2015 (41)	Brazil	RCT, SC, P, SB, TAU-C	hospital inpatient unit	50	40.3 ± 10.95	37/13	NR, patients with contraindications to exercise excluded	aerobic exercise + TAU	16.5 kcal/kg/week aerobic exercise; 3 sessions/week; ~42–43 min/session	trained research staff (physiologist)	HAM-D ≥25	TAU	Individual	Active
Kinser et al., 2014 (44)	USA	RCT, SC, P, NB, non- AC	Urban community	27	38.9 ± 12.6	27/0	Any other psychiatric conditions were extracted	Yoga	Weekly 75-min group sessions + daily home practice for 8 weeks	manual-guided	PHQ-9 ≥10 at baseline	HE	Group	Active
Yeung et al., 2012 (42)	USA	RCT, SC, P, SB,	Community-based	39	55 ± 10	30/9	Any other psychiatric conditions were extracted	Tai Chi	1 hr/session × 2 sessions/week × 12 weeks + home practice, 12-weeks	Tai Chi experienced instructor	HAM-D17 ≥12	Waitlist control	Group	Active & non-active
Mota-Pereira et al., 2011 (47)	Portugal	RCT, SC, P, NB, AC	outpatients	33	NR	NR	NR	Moderate aerobic exercise	30–45 min/day, 5 days/week × 12 weeks; 1 supervised, walk/week, 12-weeks	Weekly supervision by research team	HAM-D17 ≥	Usual pharmacotherapy only	Individual	Non-active
Lavretsky et al., 2011 (39)	USA	RCT, SC, P, DB, AC	Outpatient	73	70.57 ± 7.3	45/28	Mild anxiety, apathy	TCC + escitalopram (10–20 mg/day)	2 hours per week × 10 weeks	Certified Tai Chi Chih instructor	Overall HDRS ~17	HE + escitalopram	Group	Active
Carta et al., 2008 (23)	Italy	RCT, SC, P, NB, AC	Psychiatric Unit, Hospital	30	Range between 40-60	30/0	GAD, SP, PD	PA + pharmacotherapy (antidepressants)	60 min/session × 2/week × 32 weeks, 8-months	Skilled instructor & supervised by physician and psychologist	HAM-D > 13	Pharmacotherapy only; same antidepressant classes	Group	Active

PA, Physical Activity; TCC, Tai Chi Chih; NB, No Blinding; HE, Health-education; SB, Single Blind; LY, Laughter Yoga; HEW, Health Education and Wellness; MC, Multi Centre.

### 3.3 RoB assessment

**Figure 2:**
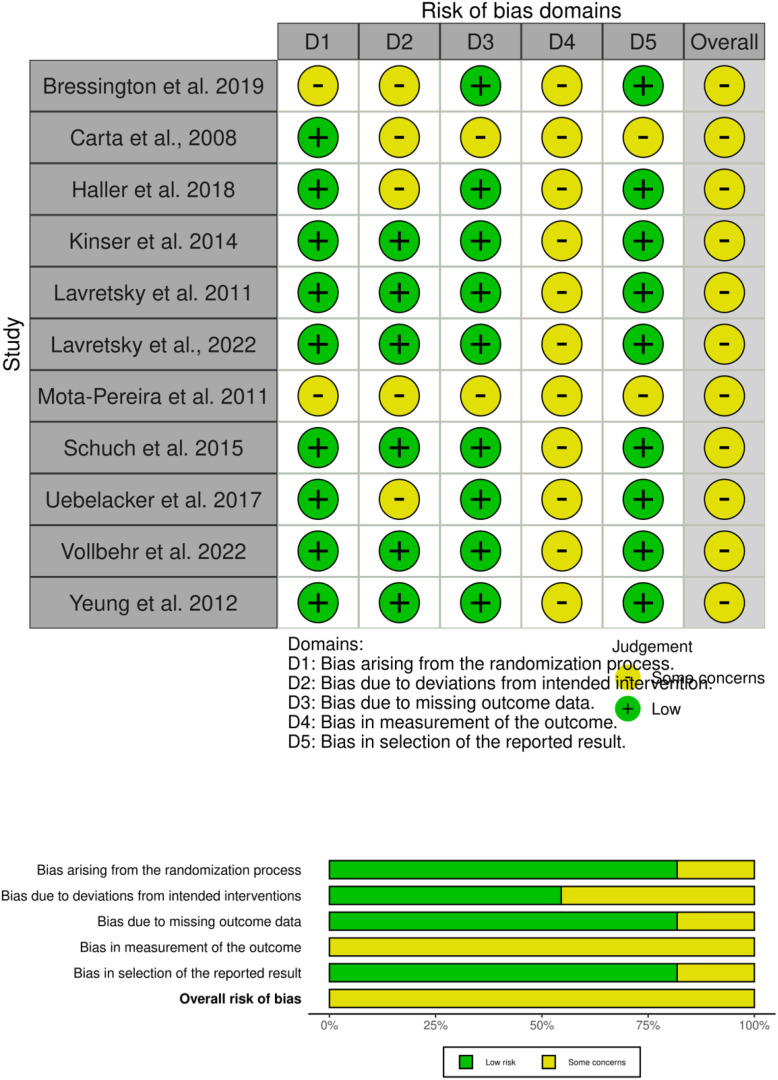
Risk of bias assessment of all included studies. The traffic-light plot (top) presents the domain-level judgments for each individual study, while the summary plot (bottom) provides an overall distribution of risk of bias across all included studies.

The risk of bias assessment of the included trials is shown in **Figure 2**. In total, all 11 studies were judged with some degree of some concerns (23, 38–47). For D1 (randomization process), nine studies were considered low risk (23, 39–46), while two were rated as having some concerns (38, 47). For D2 (deviations from intended interventions), six studies were assessed as low risk (39–44, 46) and five as some concerns (23, 38, 45–47). For D3 (missing outcome data), nine studies were judged to be at low risk (38–46) and other two studies had some concerns (23, 47). For D4 (measurement of outcomes), all 11 studies were rated as having some concerns, primarily due to assessor blinding issues or reliance on self-reported HR-QoL instruments (23, 38–47). For D5 (selection of the reported result), two studies assessed in this domain were judged as having some concerns (23, 47) and other had low risk (38–46).

### 3.4 Qualitative results

#### 3.4.1 Perceived quality findings

Across studies, participants consistently reported high satisfaction, strong adherence, and positive perceptions of intervention quality and delivery. In Vollbehr et al., 2022, post-intervention evaluations demonstrated high ratings for the MYI training, online module, and instructor, supported by fidelity checks showing excellent adherence (postures: 97.7%; mindful cues: 95.6%) and strong instructor evaluations (attitude: 4.50/5; mindful cues: 4.14/5; competence: 4.04/5; yoga quality: 3.84/5) (43). Lavretsky et al., 2022 observed high adherence (TCC: 82.3%; HEW: 79.2%) (40), while Bressington et al., 2019 found strong acceptability through CSQ-8 scores (α = 0.83–0.93) and themes of relaxation and social connectedness (38). Participants in Haller et al., 2018 rated the web-based program as highly feasible, user-friendly, and satisfactory, with only minor orthopaedic complaints reported (45). Similarly, Uebelacker et al., 2017 reported excellent fidelity for both yoga (95% content; 94% teaching) and HLW (97% content; 95% teaching) (46). Long-term interviews in Kinser et al., 2014 revealed sustained acceptability, with participants citing benefits such as coping skills, relaxation, and empowerment (44). In Yeung et al., 2012, most participants expressed positive expectations toward Tai Chi, achieving 73% adherence (≥65% sessions) (42). High adherence and engagement were also evident in Mota-Pereira et al., 2011 (47) (91%) and Carta et al., 2008 (23) (71%), while Lavretsky et al., 2011 found both TCC and HE credible, and satisfying (39).

#### 3.4.2 Potential comparator findings

Several studies identified mediators and predictors that could influenced intervention outcomes. In Vollbehr et al., 2022, reliable measures across T0–T3 (rumination α = .92–.97; self-criticism α = .88–.93; self-compassion α = .93–.94; intolerance of uncertainty α = .84–.91; body awareness α = .82–.87; mindfulness α = .76–.98) highlighted cognitive-emotional mechanisms are linked to perceived quality (43). Lavretsky et al., 2022 reported resilience and lower baseline depression severity predicting remission at 3 and 6 months, with male sex associated with higher remission odds (40). In Bressington et al., 2019, qualitative themes suggested stress reduction, relaxation, and social connectedness as affective mediators of depressive symptom change (38). Haller et al., 2018 found that early engagement, consistent participation, and increased habitual activity predicted greater depressive symptom improvement (45). Uebelacker et al., 2017 linked greater yoga practice to lower depression at follow-up, indicating dose-response effects (46). Sustained practice was similarly associated with lower depression in Kinser et al., 2014 (44). Lavretsky et al., 2011 proposed that mind–body regulation and stress resilience mediated improvements in QoL and depressive symptoms (39).

#### 3.4.3 Health-related quality of life (HR-QoL) findings

HR-QoL outcomes were mixed but generally improved over time. Vollbehr et al., 2022 observed no significant group differences in impairment, satisfaction with health, or purpose in life, though overall scores improved over time (43). Lavretsky et al., 2022 found significant within-group HR-QoL improvements in both TCC and HEW arms without between-group differences (40). Bressington et al., 2019 reported trends toward improved self-reported physical and mental health at post-test and 3-month follow-up, though non-significant vs TAU (38). Haller et al., 2018 showed significant gains in emotional well-being and social functioning, but non-significant total HR-QoL change (45). Uebelacker et al., 2017 found yoga participants reported better overall HR-QoL across all non-baseline timepoints, maintained through 3- and 6-month follow-ups (46). Schuch et al., 2015 observed significant improvements in physical and psychological WHOQOL-BREF domains, while social and environmental domains remained unchanged (41). Kinser et al., 2014 found HR-QoL improved over time across groups, with long-term mental HR-QoL advantages in yoga participants at 52 weeks (44). Yeung et al., 2012 reported HR-QoL gains in the Tai Chi group, though non-significant vs control (42). In Mota-Pereira et al., 2011, remitted patients achieved QoL comparable to healthy individuals; exercise improved physical and social domains only (47). Lavretsky et al., 2011 found TCC produced greater physical functioning improvement vs HE (39), while Carta et al., 2008 showed significant physical health domain gains over 8 months, with other domains unchanged (23).

#### 3.4.4 Other Psychiatric Outcomes

Secondary psychiatric outcomes showed broad improvement across interventions. Vollbehr et al., 2022 found overall improvement in psychological functioning across time, without between-group differences (43). Lavretsky et al., 2022 observed depression remission over 6 months, with no significant differences between TCC and HEW (40). Bressington et al., 2019 reported within-group reductions in depression, anxiety, and stress for LY participants, but no significant between-group effects (38). Haller et al., 2018 demonstrated depressive symptom reduction linked to adherence, without significant group differences (45). Uebelacker et al., 2017 found yoga reduced depressive symptoms and improved social and role functioning vs HLW (46). Schuch et al., 2015 observed greater depressive symptom reduction in exercise vs TAU (41). Kinser et al., 2014 reported reduced depression and rumination at 52 weeks, with improvements in stress and anxiety across groups (44). Yeung et al., 2012 found no significant group differences in depressive symptoms (42). Mota-Pereira et al., 2011 reported significantly greater reductions in depression and anxiety in the exercise group (47). Lavretsky et al., 2011 showed combined TCC+SSRI treatment yielded superior depression severity reduction vs HE+SSRI (39). Finally, Carta et al., 2008 demonstrated improved depression severity and global functioning in the PA group vs control (23).

### 3.5 Synthesis of results

#### 3.5.1 Overall HR-QoL findings

We evaluated the impact of exercise on HR-QoL at two time points: immediately after the intervention and at the final follow-up following the intervention.

**Figure 3:**
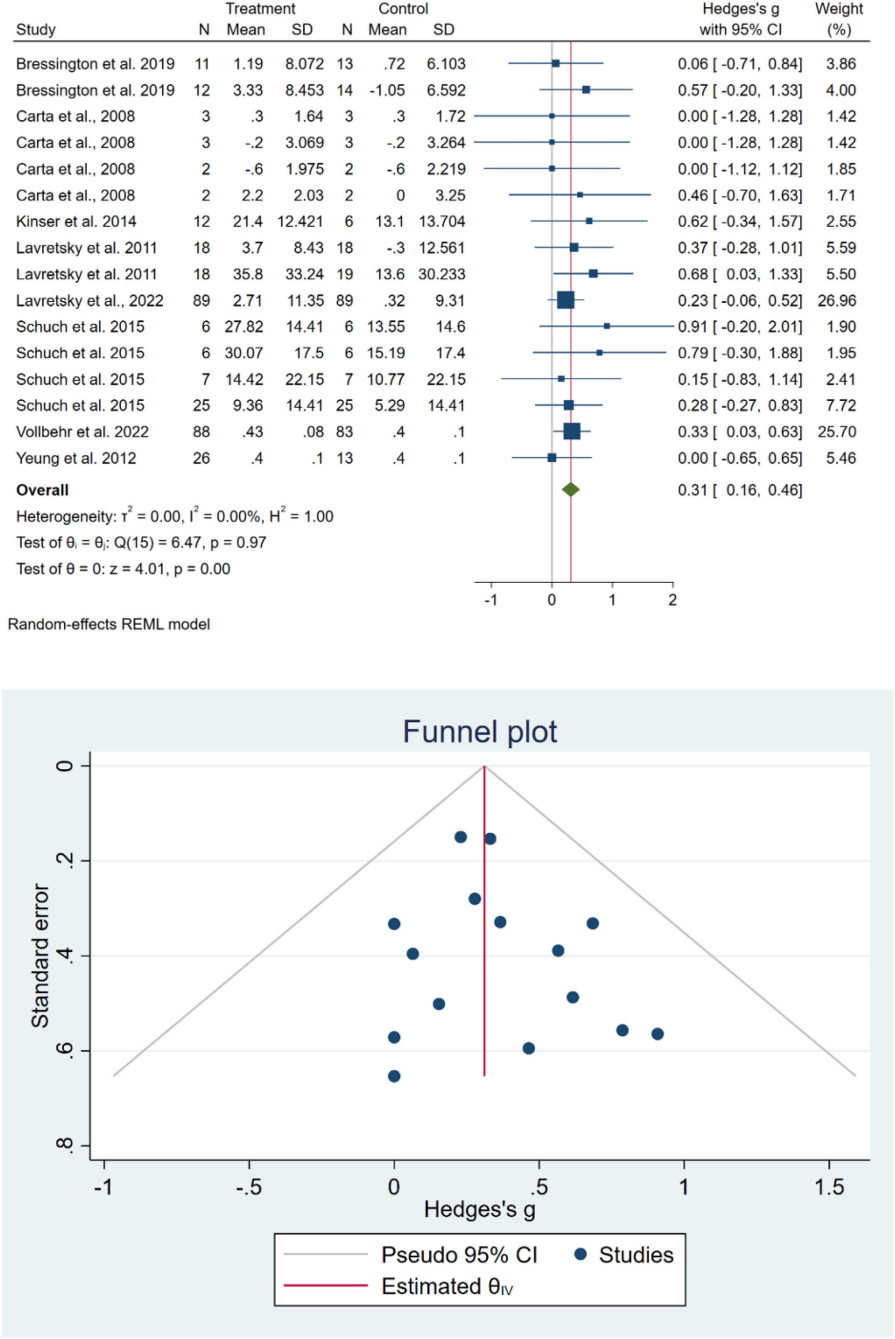
Meta-analysis of HR-QoL outcomes immediately after the end of treatment (upper panel) and assessment of publication bias using funnel plot and statistical tests (lower panel).

At the immediate post-treatment assessment, pooled results from eight studies (23, 38–44) encompassing 16 effect sizes showed that exercise led to a significant improvement in HR-QoL compared with the control group (SMD = 0.31 [0.16, 0.46], I² = 0.00%) (**Figure 3**). Sensitivity analysis indicated that excluding any single study did not alter the overall pooled effect (**Supplementary Material**, **Supplementary Figure 1**). The funnel plot revealed a symmetric distribution of studies, further supported by Egger’s (p = 0.67) and Begg’s (p = 0.79) tests, suggesting no publication bias (**Figure 3**). Subgroup analysis showed no significant differences between groups (**Supplementary Material**, **Supplementary Figure 2**).

**Figure 4:**
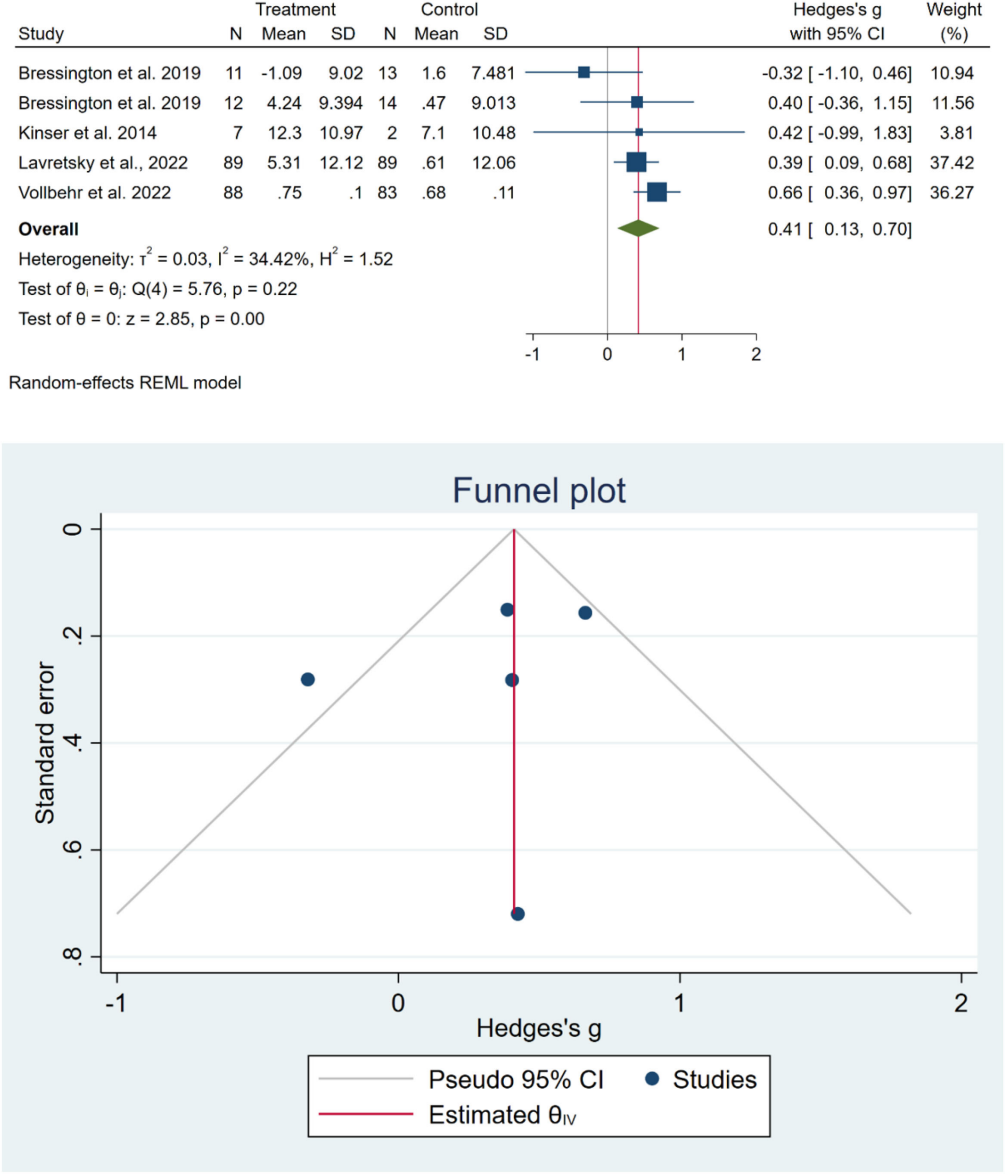
Meta-analysis of HR-QoL outcomes at the last follow-up after treatment (upper panel) and assessment of publication bias using funnel plot and statistical tests (lower panel).

At follow-up, pooled analysis of four studies (38, 40, 43, 44) with five effect sizes indicated that exercise was associated with a significant improvement in HR-QoL compared with the control group (SMD = 0.41, 95% CI: 0.13 to 0.70; I² = 34.42%) (**Figure 4**). However, sensitivity analysis demonstrated that removing Lavretsky et al. (40) the study shifted the pooled effect (SMD = 0.36, 95% CI: -0.12, 0.84) (**Supplementary Material**, **Supplementary Figure 3**). The funnel plot was symmetric, and both Egger’s (p = 0.30) and Begg’s (p > 0.99) tests indicated no evidence of publication bias (**Figure 4**). Subgroup analysis likewise revealed no significant differences between groups (**Supplementary Material**, **Supplementary Figure 4**).

#### 3.5.2 Domains of the HR-QoL

**Table 4** summarizes the analysis of the domains of HR-QoL, comparing the effects of exercise interventions to control conditions. The physical domain refers to aspects such as physical functioning, energy/fatigue, pain, and general health status, as assessed by validated HR-QoL instruments (e.g., SF-36, WHOQOL-BREF). The overall effect across three studies (40, 42, 43) showed significant improvement favoring exercise (SMD = 0.25, 95% CI [0.05, 0.45], p = 0.01) with no observed heterogeneity (I² = 0%). In the physical domain, four studies (23, 38, 39, 41) demonstrated significant benefit of exercise over control (SMD = 0.50, 95% CI [0.10, 0.91], p = 0.02), although heterogeneity was moderate (I² = 48.18%). The psychological domain showed the strongest effect, with four studies (23, 38, 41, 44) indicating a moderate and statistically significant improvement in the exercise groups (SMD = 0.58, 95% CI [0.24, 0.92], p < 0.001) and no heterogeneity (I² = 0%). The two studies (39, 41) which explored the emotional domain also demonstrated a moderate, significant advantage of exercise compared to control (SMD = 0.51, 95% CI [0.11, 0.91], p = 0.01) with low heterogeneity (I² = 21.03%). In contrast, the social domain showed no significant difference between exercise and control groups (SMD = 0.11, 95% CI [–0.34, 0.57], p = 0.63) (23, 41), and heterogeneity was absent (I² ~ 0%) (**Supplementary Material**, **Supplementary Figures 5****–****14**).

**Table 4:** Pooled meta-analysis of HR-QoL domains comparing exercise interventions with control conditions.

Domain	N of effect sizes	SMD [CI]	I^2^	P-value
Overall	3	0.25 [0.05, 0.45]	0.00%	0.01
Physical	4	0.50 [0.10, 0.91]	48.18%	0.02
Psychological	4	0.58 [0.24, 0.92]	0.00%	0.00
Social	2	0.11 [-0.34, 0.57]	0.00%	0.63
Emotional	2	0.51 [0.11, 0.91]	21.03%	0.01

### 3.6 Certainty of evidence (GRADE)

**Table 2** presents the GRADE assessment of the main outcomes. The certainty of evidence was moderate for HR-QoL total immediately post-intervention (8 studies, n = 637; SMD = 0.31, 95% CI 0.16 to 0.46) and for overall HR-QoL at follow-up (4 studies, n = 408; SMD = 0.41, 95% CI 0.13 to 0.70). Moderate certainty was also observed for the overall/global and psychological domains. The certainty was low for the physical, social, and emotional domains, mainly due to imprecision and small sample sizes. Overall, evidence was primarily downgraded for risk of bias, while publication bias was not considered a major concern.

## 4 Discussion

This meta-analysis involving eleven randomized controlled trials reveals that exercise-based interventions lead to improvement in the HR-QoL of patients suffering from MDD. Although a small but significant impact has been observed at post-intervention stage, it is consistent and strong in all sensitivity analyses. On the other hand, significant impact was observed at the follow-up stage, but this is questionable because there were very few studies, and it also became insignificant when the influence of a single study was stripped off. It can be concluded that the impact of exercise interventions on quality of life occurs both in the course of treatment and possibly beyond (48, 49).

Such results are compatible with earlier research demonstrating short-term HR-QoL improvement through exercise therapy. For instance, Tai Chi in combination with pharmacotherapy is linked to increased vitality and role function. Furthermore, yoga-based programs have been shown to result in better HR-QoL concerning psychological functioning in patients undergoing standard therapy (39, 44). Collectively, these results indicate that exercise can serve as an efficient short-term facilitator of not only psychological wellbeing but also general quality of life (50). In turn, there is little data on sustained long-term results, which may be explained by difficulties with adhering to exercise programs. It is also possible that after exercise programs, there is no further assistance provided, resulting in a decline of effects. The same has been observed in other types of populations (51).

The improvements in HR-QoL were most pronounced immediately after the intervention. This suggests that exercise may act as a short-term enhancer of well-being in individuals with MDD. However, the limited number of studies reporting follow-up data and variability in follow-up durations reduce confidence in conclusions regarding sustained benefits.

Several factors may explain the lack of sustained effects. These include declining adherence over time, loss of structured support, and insufficient intervention duration to establish long-term behavioural change. In addition, individuals may return to sedentary lifestyles after the intervention, reducing continued exposure to the benefits of exercise (52).

Some studies suggest that sustained engagement may support longer-term benefits. For example, continued yoga practice has been associated with maintained improvements in HR-QoL, potentially due to enhanced self-regulation. In contrast, shorter-duration interventions, such as brief laughter yoga programs, may not produce lasting effects (38, 44).

Domain-level analyses provide important insights into how different components of HR-QoL respond to exercise interventions. The findings suggest that psychological and emotional domains are particularly sensitive to exercise, whereas social HR-QoL appears less responsive. The psychological domain demonstrated the strongest and most consistent improvements. This aligns with previous meta-analytic evidence showing that exercise reduces depressive symptoms and enhances cognitive-emotional functioning (53, 54).

These effects may be explained by both psychological and biological mechanisms. Exercise has been shown to improve self-efficacy, reduce maladaptive rumination, and enhance stress regulation. In addition, emerging evidence suggests that physical activity promotes neuroplasticity through increased brain-derived neurotrophic factor (BDNF), reduces systemic inflammation, and modulates hypothalamic–pituitary–adrenal (HPA) axis activity. These mechanisms are closely linked to the pathophysiology of MDD and may contribute to improvements in both mood and perceived quality of life (55, 56).

Additionally, the observed improvements in the physical domain support the close relationship between physical and mental health. Enhanced physical functioning, increased energy levels, and reduced somatic symptoms may contribute to improved overall life satisfaction.

In contrast, social HR-QoL did not improve significantly. This may reflect the individualistic nature of many exercise interventions, which provide limited opportunity for social interaction or role restoration (57). Studies incorporating group-based formats have reported subjective gains in connectedness, suggesting that socially embedded exercise programs may be necessary to impact this domain (38). Therefore, tailoring to a domain-specific example that embodies either group cohesion or peer support may enhance social benefits.

Beyond HR-QoL, emerging evidence suggests that exercise may also improve cognitive and executive functioning in individuals with MDD. Recent meta-analyses have reported benefits across domains such as attention, memory, processing speed, and inhibitory control. In addition, exercise appears to have antidepressant effects, with moderate levels of activity associated with optimal symptom reduction (58) (25).

However, our meta-analysis did not quantitatively evaluate secondary outcomes, although numerous studies within our review examined depressive symptoms, anxiety, and rumination. For example, in two of the studies (Mota-Pereira et al., 2011; Schuch et al., 2015) the authors reported significant reductions in overall depression severity after aerobic exercise and structured exercise (41, 47), whereas Uebelacker et al. (2017) and Kinser et al. (2014) reiterated the importance of improved rumination and improved stress management within a yoga intervention (44, 46). It is reasonable to believe that these secondary outcomes are mechanisms for HR-QoL to improve, and are likely to be embellished by a domain-specific modifiable example, particularly in the psychological and emotional domains.

Overall, the findings suggest a potential pathway in which exercise reduces depressive symptoms and maladaptive cognitive patterns, which in turn improves perceived quality of life. This interpretation is supported by evidence indicating that greater engagement in exercise is associated with larger reductions in symptoms (44, 46).

These findings are consistent with previous systematic reviews and meta-analyses examining the effects of exercise in individuals with MDD. For example, recent network meta-analyses have demonstrated that structured exercise programs can improve depressive symptoms across various modalities (35). Likewise, Krogh et al., 2017 conducted a systematic review with meta-analysis and trial sequential analysis examining the effect of exercise on depression or depressive symptoms, and they reported a small to moderate effect for exercise on depressive symptoms. However, the quality of evidence was low, and they did not observe a significant effect on other outcomes such as HR-QoL (59).

From a clinical perspective, these findings support the use of exercise as a feasible and low-cost adjunct to standard treatment for MDD. Exercise interventions may lead to meaningful improvements in HR-QoL within a relatively short time frame (60).

Overall, the present findings are broadly consistent with previous meta−analyses reporting beneficial effects of exercise on depressive symptoms and psychological functioning. However, according to the GRADE framework applied in the current review, the certainty of evidence ranged from low to moderate across outcomes. While moderate certainty was observed for overall HR−QoL and psychological domains, the physical, social, and emotional domains were rated as low certainty due to imprecision and limited sample sizes. Therefore, the results should be interpreted cautiously, particularly when generalizing the findings to broader clinical populations.

To our knowledge, this is among the first and only meta-analysis to synthesize randomized controlled trials assessing the impact of exercise on HR-QoL in adults with MDD, distinguishing it from previous reviews of exercise and prior studies primarily examining depressive symptoms and cognition (e.g., Ren et al., 2024, Tian et al., 2024) (25, 31). The present study highlights a considerable contribution to exercise literature, providing a more nuanced examination of domains of psychological, emotional, and physical HR-QoL on exercise. Using RCT data only increased internal validity and low heterogeneity across the pooled analyses strengthened the results.

The methodological strength of the present meta−analysis is the careful handling of multiple effect sizes derived from the same study. To avoid unit−of−analysis errors and within−study dependency, shared sample sizes were divided across relevant HR−QoL domains when multiple outcomes from the same trial contributed to a single analysis. This approach follows Cochrane methodological guidance and reduces the risk of double counting participants in pooled estimates.

However, the GRADE approach enhances the interpretation of the results. Moderate certainty existed in terms of HR-QoL immediately after intervention, at follow-up, and in the global domain of HR-QoL and psychological domain. These findings show that exercise is effective in improving HR-QoL in adults with MDD. On the other hand, certainty was low regarding the effects on the physical, social, and emotional domains of HR-QoL since most of the studies used had small sample sizes and were imprecise. Most of the findings were additionally downgraded in regards to risk of bias due to issues with measurements and poor blinding.

To our knowledge, this is among the first and only meta-analysis to synthesize randomized controlled trials assessing the impact of exercise on HR-QoL in adults with MDD, distinguishing it from previous reviews of exercise and prior studies primarily examining depressive symptoms and cognition (e.g., Ren et al., 2024, Tian et al., 2024) (25, 31). The present study highlights a considerable contribution to exercise literature, providing a more nuanced examination of domains of psychological, emotional, and physical HR-QoL on exercise. Using RCT data only increased internal validity and low heterogeneity across the pooled analyses strengthened the results. Overall, the present analysis moves the field forward by providing strong preliminary support for exercise as a generalizable, adjunctive treatment to promote HR-QoL in adults with MDD and provides a good perspective for future research to further assess maintenance and potential long-term integration of exercise.

One significant strength of the current review is the rigorous methodology and transparent process used in the analysis. The restriction of the current meta-analysis to randomized controlled trials among adult patients with clinically confirmed MDD enhances the validity and clinical utility of the results obtained. Another significant methodological strength of the current meta-analysis is the accurate computation of multiple effect sizes that may have been drawn from the same study. To prevent any unit-of-analysis error and intra-study dependence, common sample sizes for each study were apportioned among each outcome in relation to HR-QoL. This methodology is in accordance with the guidelines by Cochrane and minimizes the risk of double counting of subjects in pooled analyses. Moreover, the implementation of the GRADE system and the use of a Summary of Findings table enhance the transparency and clinical significance of the systematic review.

It is essential to identify some important issues while interpreting the findings from the study. First, even though only RCTs were selected to reduce potential bias, some heterogeneity among included studies may exist due to the difference in the intervention intensity, duration, supervision, and compliance measures. Secondly, only a handful of trials mentioned follow-up results, which made the assessment of sustainability of HR-QoL improvement less confident. Besides, baseline differences in depression severity, comorbidity, and medications used can also impact the results obtained through the meta-analysis. Thirdly, it is worth mentioning that this systematic review was not registered prior to the analysis, and hence, its transparency could be increased by adding this step. Lastly, it is important to note that studies published in Chinese or any other language besides English were not included in the analysis, potentially missing studies focused on exercises like Tai Chi, yoga, or qigong.

Research in the future needs to go beyond the question of effectiveness alone and focus on properly powered studies using appropriate follow-ups along with standardized measurement tools for HR-QoL and a specified period of time during which data will be collected. Researchers also need to consider adopting a behavior maintenance model to explain how exercise can continue even after the supervision of a professional is withdrawn, in accordance with current guidelines for conducting trials involving complex interventions. Moreover, future research needs to focus on identifying any moderators, including depression severity, gender, age, comorbidities, and mode of exercise (61).

## 5 Conclusion

Exercise treatment can positively influence the HR-QoL of individuals with MDD, especially in the early stages and especially for physical and psychological functioning. While pooling results from follow-ups showed some benefit, it was based on a small body of literature that was somewhat less convincing upon sensitivity testing, hence leaving the long-term effect of exercise on HR-QoL unexplored. In addition, there is no strong evidence yet that would point out which exercise type is better than others. This conclusion must be considered taking into account the relatively small number of trials involved, the heterogeneity of control conditions, and the lack of certainty on the measurement instruments used. There is an urgent need for conducting high-quality, well-powered randomized clinical trials.

## Data Availability

The original contributions presented in the study are included in the article/**Supplementary Material**. Further inquiries can be directed to the corresponding author.
