# Supplementary material for: Physical Activity interventions on health-related quality of life (HR-QoL) in major depressive disorder (MDD): a systematic review and meta-analysis of randomized controlled trials
Source: Front Psychiatry. 2026 May 22;17:1806489. doi: 10.3389/fpsyt.2026.1806489 (PMC13236921; doi:10.3389/fpsyt.2026.1806489)
Supplement: Supplementary file 1 [file Supplementaryfile1.docx]

**Search strategy**

**Depression**

Depression* OR “depressive symptom*” OR “depressive disorder*” OR “major depressive disorder” OR Melancholia*

**Quality**

“quality of life” OR “health related quality of life” OR “HRQoL” OR “life quality”

**Exercise**

Exercise* OR exercising OR yoga OR “tai-chi” OR “tai chi” OR “tai ji” OR qigong OR “qi gong” OR “Physical Activity”

**RCT**

"double-blind" OR "random* assigned" OR control

**Databases**

1. ***PubMed: 2642 results***

(((((((Depression*[Title/Abstract]) OR ("depressive symptom*"[Title/Abstract])) OR ("Depression"[Mesh])) OR ("Depressive Disorder"[Mesh])) OR ("depressive disorder*"[Title/Abstract])) OR ("major depressive disorder"[Title/Abstract])) OR (Melancholia*[Title/Abstract])) OR ("Depressive Disorder, Major"[Mesh])

(((("quality of life"[Title/Abstract]) OR ("health related quality of life"[Title/Abstract])) OR ("HRQoL"[Title/Abstract])) OR ("life quality"[Title/Abstract])) OR ("Quality of Life"[Mesh])

(((((((((Exercise*[Title/Abstract]) OR (exercising[Title/Abstract])) OR (yoga[Title/Abstract])) OR ("tai-chi"[Title/Abstract])) OR ("tai chi"[Title/Abstract])) OR ("tai ji"[Title/Abstract])) OR (qigong[Title/Abstract])) OR ("qi gong"[Title/Abstract])) OR ("Physical Activity"[Title/Abstract])) OR ("Exercise"[Mesh])

((("double-blind"[Title/Abstract]) OR ("random* assigned"[Title/Abstract])) OR (control[Title/Abstract])) OR ("Randomized Controlled Trial" [Publication Type])

1. ***Scopus: 5123 results***

TITLE-ABS-KEY(Depression*) OR TITLE-ABS-KEY(“depressive symptom*”) OR TITLE-ABS-KEY(“depressive disorder*”) OR TITLE-ABS-KEY(“major depressive disorder”) OR TITLE-ABS-KEY(Melancholia*)

TITLE-ABS-KEY( “quality of life” ) OR TITLE-ABS-KEY( “health related quality of life” ) OR TITLE-ABS-KEY( “HRQoL” ) OR TITLE-ABS-KEY( “life quality” )

TITLE-ABS-KEY(Exercise*) OR TITLE-ABS-KEY(exercising) OR TITLE-ABS-KEY(yoga) OR TITLE-ABS-KEY( “tai-chi” ) OR TITLE-ABS-KEY( “tai chi” ) OR TITLE-ABS-KEY( “tai ji” ) OR TITLE-ABS-KEY(qigong) OR TITLE-ABS-KEY( “qi gong” ) OR TITLE-ABS-KEY( “Physical Activity”)

TITLE-ABS-KEY("double-blind") OR TITLE-ABS-KEY("random* assigned") OR TITLE-ABS-KEY(control)

1. ***Web of Science: 4993 results***

TS= (Depression* OR “depressive symptom*” OR “depressive disorder*” OR “major depressive disorder” OR Melancholia*)

TS=(“quality of life” OR “health related quality of life” OR “HRQoL” OR “life quality”)

TS=(Exercise* OR exercising OR yoga OR “tai-chi” OR “tai chi” OR “tai ji” OR qigong OR “qi gong” OR “Physical Activity”)

TS=("double-blind" OR "random* assigned" OR control)

1. ***Cochrane central: 6721 results***

(Depression*):ti,ab,kw OR (“depressive symptom*”):ti,ab,kw OR (“depressive disorder*”):ti,ab,kw OR (“major depressive disorder”):ti,ab,kw OR (Melancholia*):ti,ab,kw

(“quality of life”):ti,ab,kw OR (“health related quality of life”):ti,ab,kw OR (“HRQoL”):ti,ab,kw OR (“life quality”):ti,ab,kw

(Exercise*):ti,ab,kw OR (exercising):ti,ab,kw OR (yoga):ti,ab,kw OR (“tai-chi”):ti,ab,kw OR (“tai chi”):ti,ab,kw OR (“tai ji”):ti,ab,kw OR (qigong):ti,ab,kw OR (“qi gong”):ti,ab,kw OR (“Physical Activity”):ti,ab,kw

1. **PsycInfo: 501**

XB (“quality of life”) OR XB (“health related quality of life”) OR XB (“HRQoL”) OR XB (“life quality”)

XB (Exercise*) OR XB (exercising) OR XB (yoga) OR XB (“tai-chi”) OR XB (“tai chi”) OR XB (“tai ji”) OR XB (qigong) OR XB (“qi gong”) OR XB (“Physical Activity”)

XB (Exercise*) OR XB (exercising) OR XB (yoga) OR XB (“tai-chi”) OR XB (“tai chi”) OR XB (“tai ji”) OR XB (qigong) OR XB (“qi gong”) OR XB (“Physical Activity”)

XB (Depression*) OR XB (“depressive symptom*”) OR XB (“depressive disorder*”) OR XB (“major depressive disorder”) OR XB (Melancholia*)

**Figures**


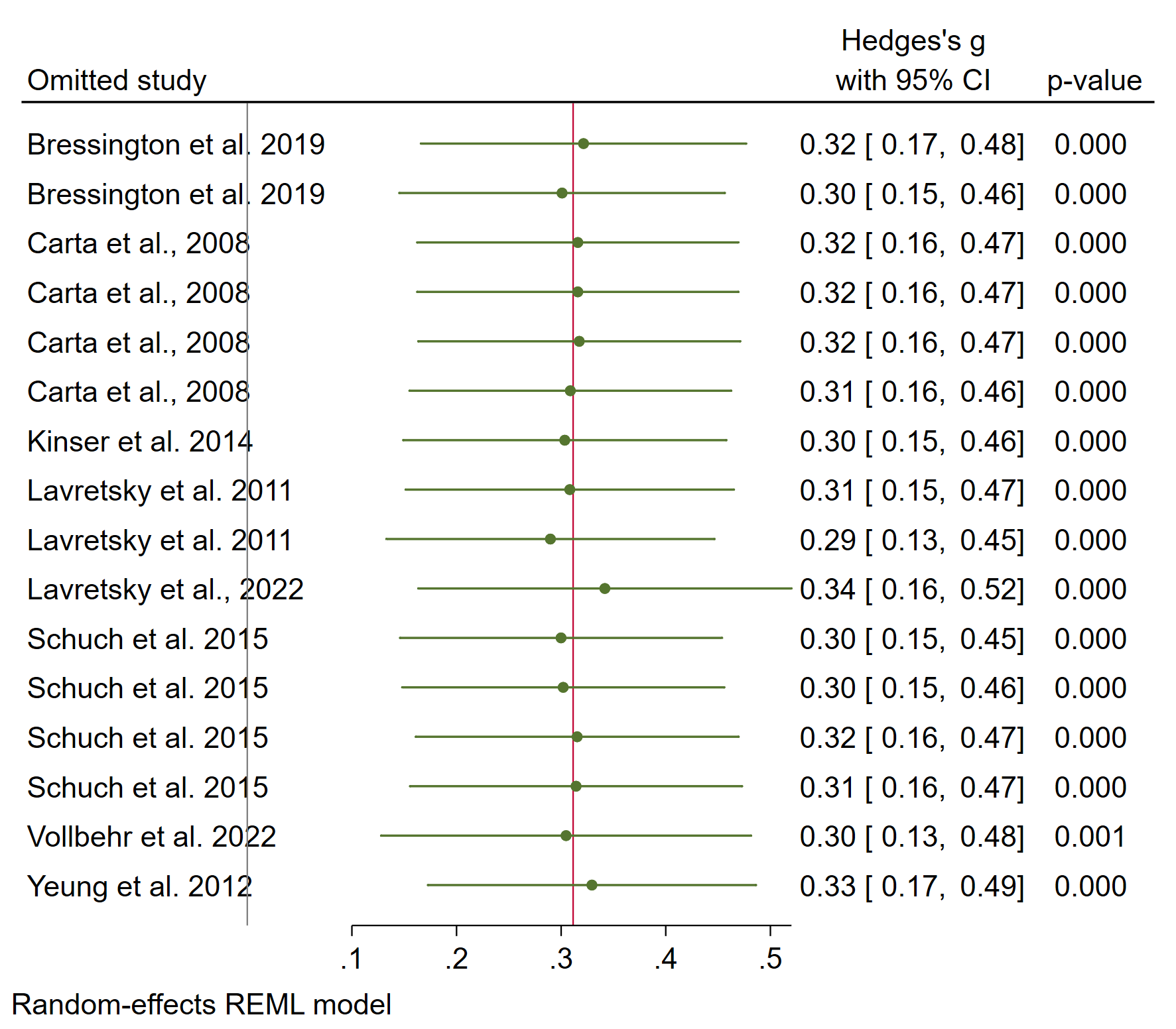


Figure S1. Sensitivity analysis of HRQoL outcomes immediately after the end of treatment.


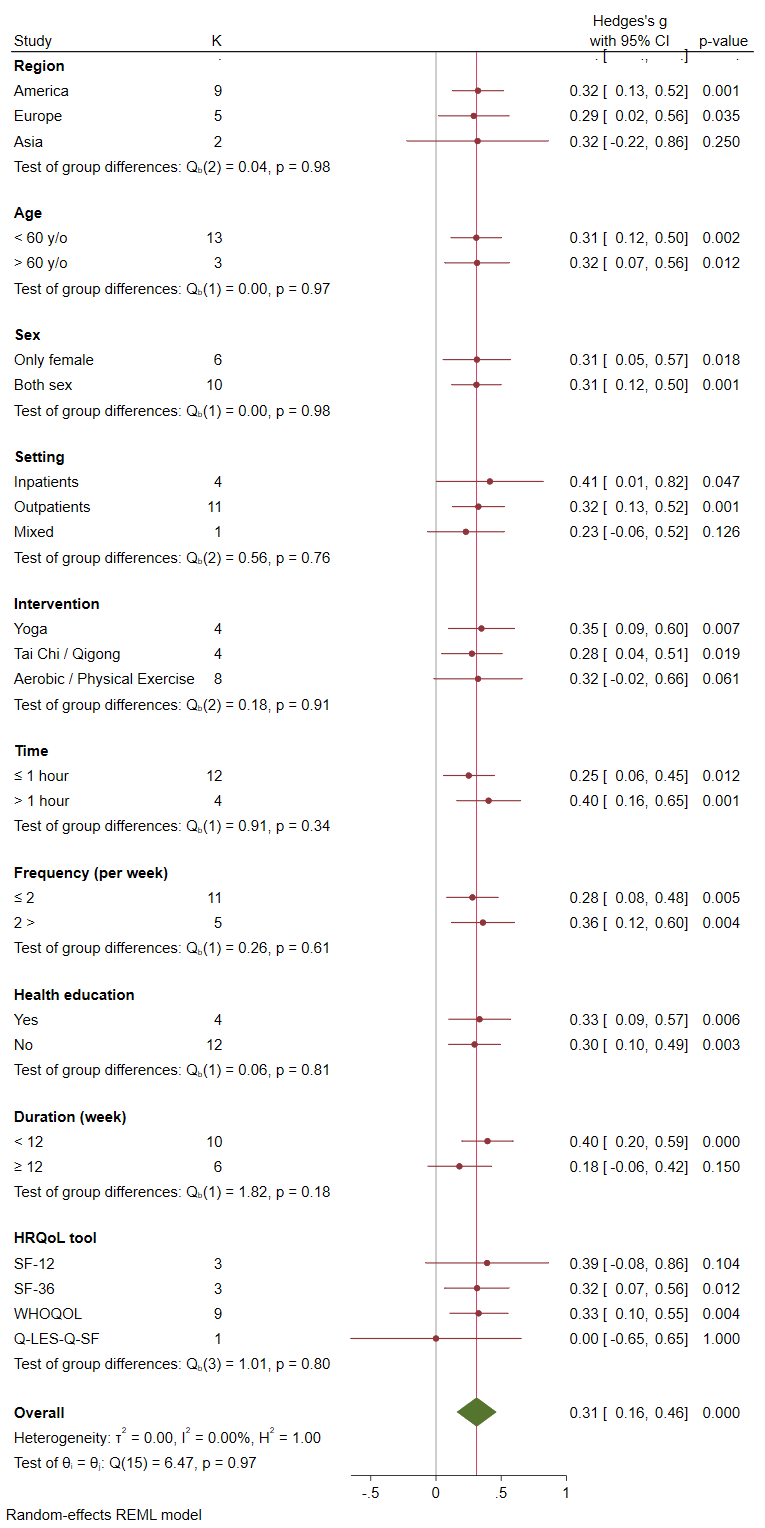


Figure S2. Subgroup analysis of HRQoL outcomes immediately after the end of treatment.


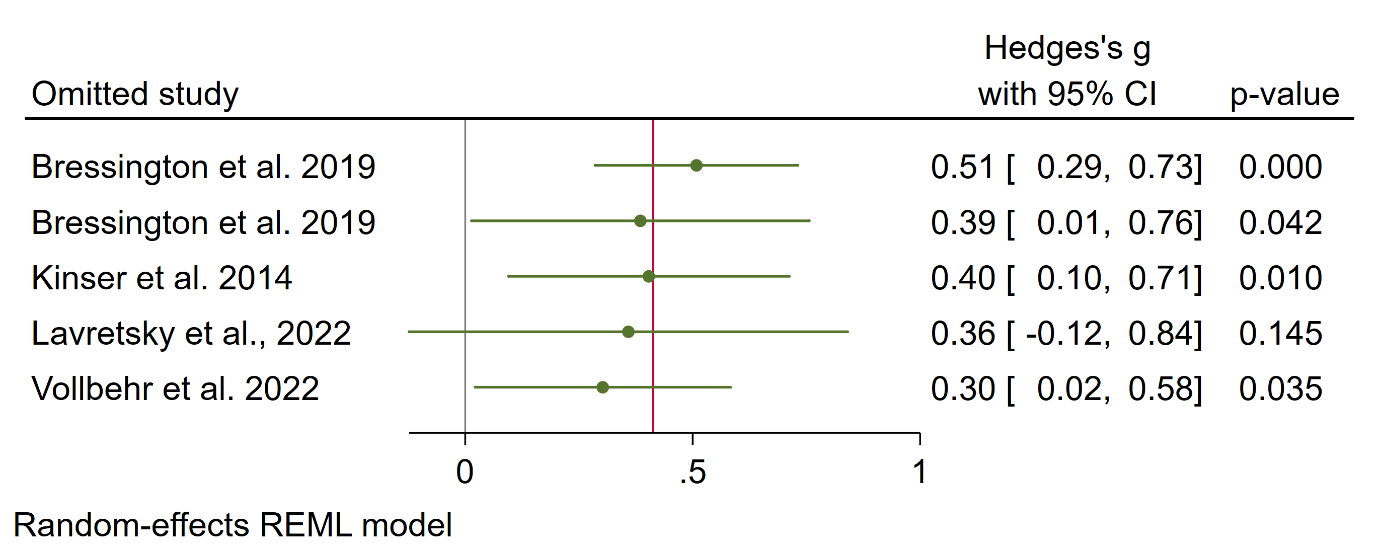


Figure S3. Sensitivity analysis of HRQoL outcomes at the last follow-up after treatment


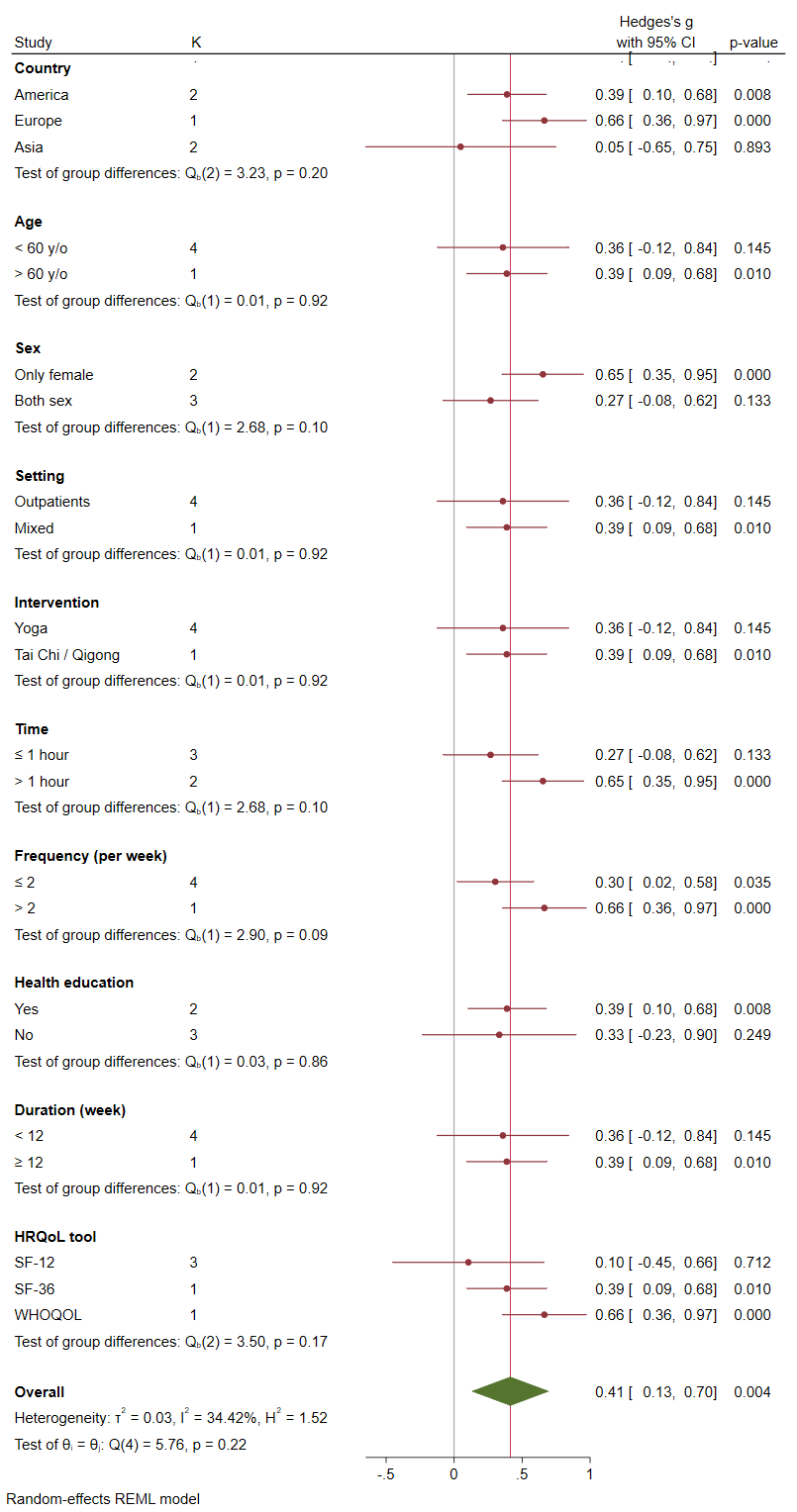


Figure S4. Subgroup analysis of HRQoL outcomes at the last follow-up after treatment


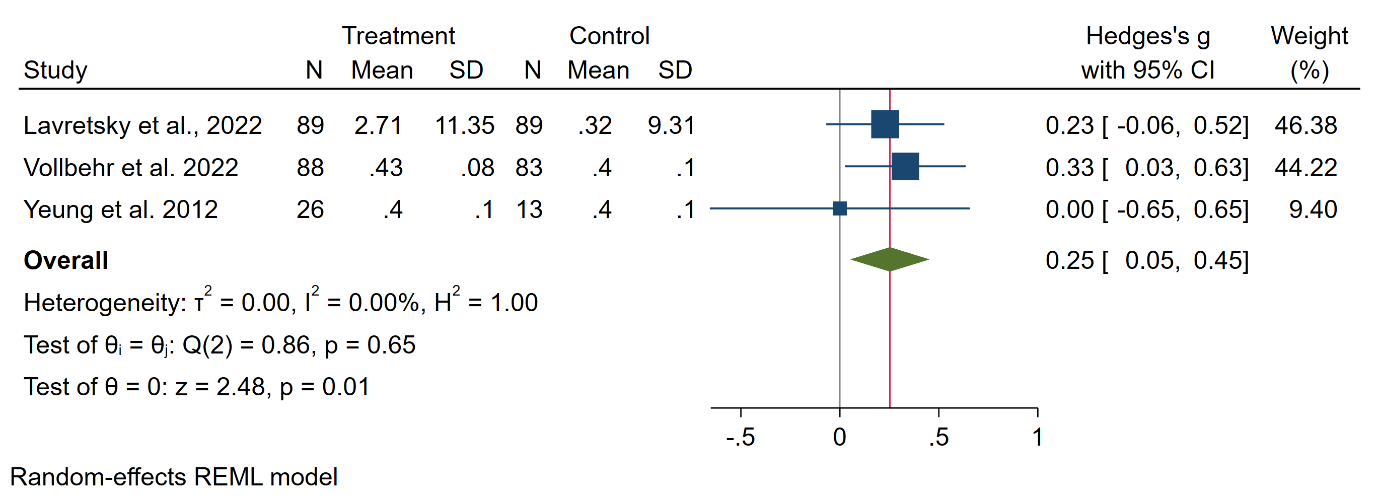


Figure S5. Meta-analysis of the effects of exercise interventions on the overall domain of HRQoL compared with control conditions.


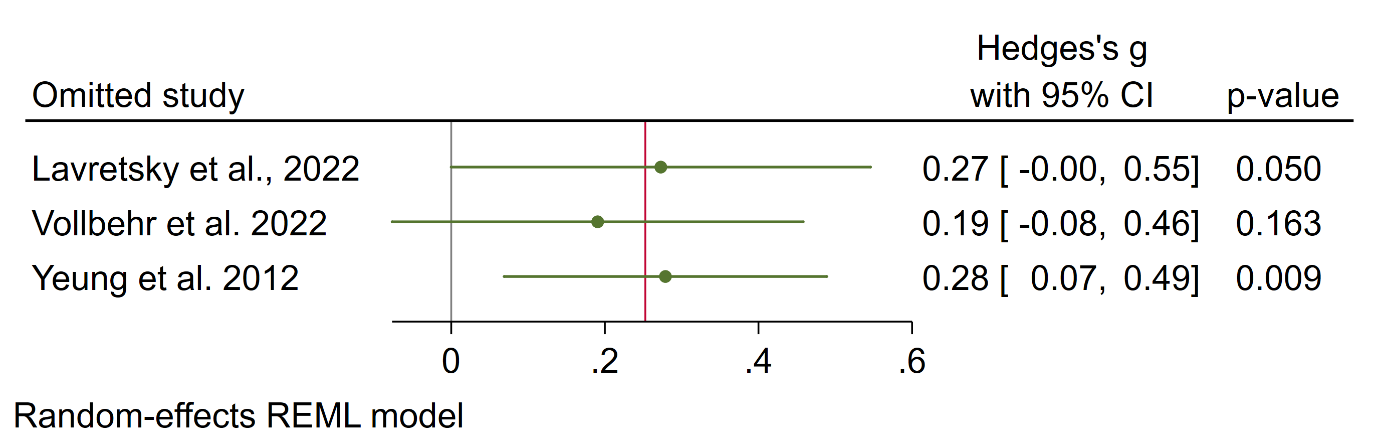
Figure S6. Sensitivity analysis of the effects of exercise interventions on the overall domain of HRQoL compared with control conditions.


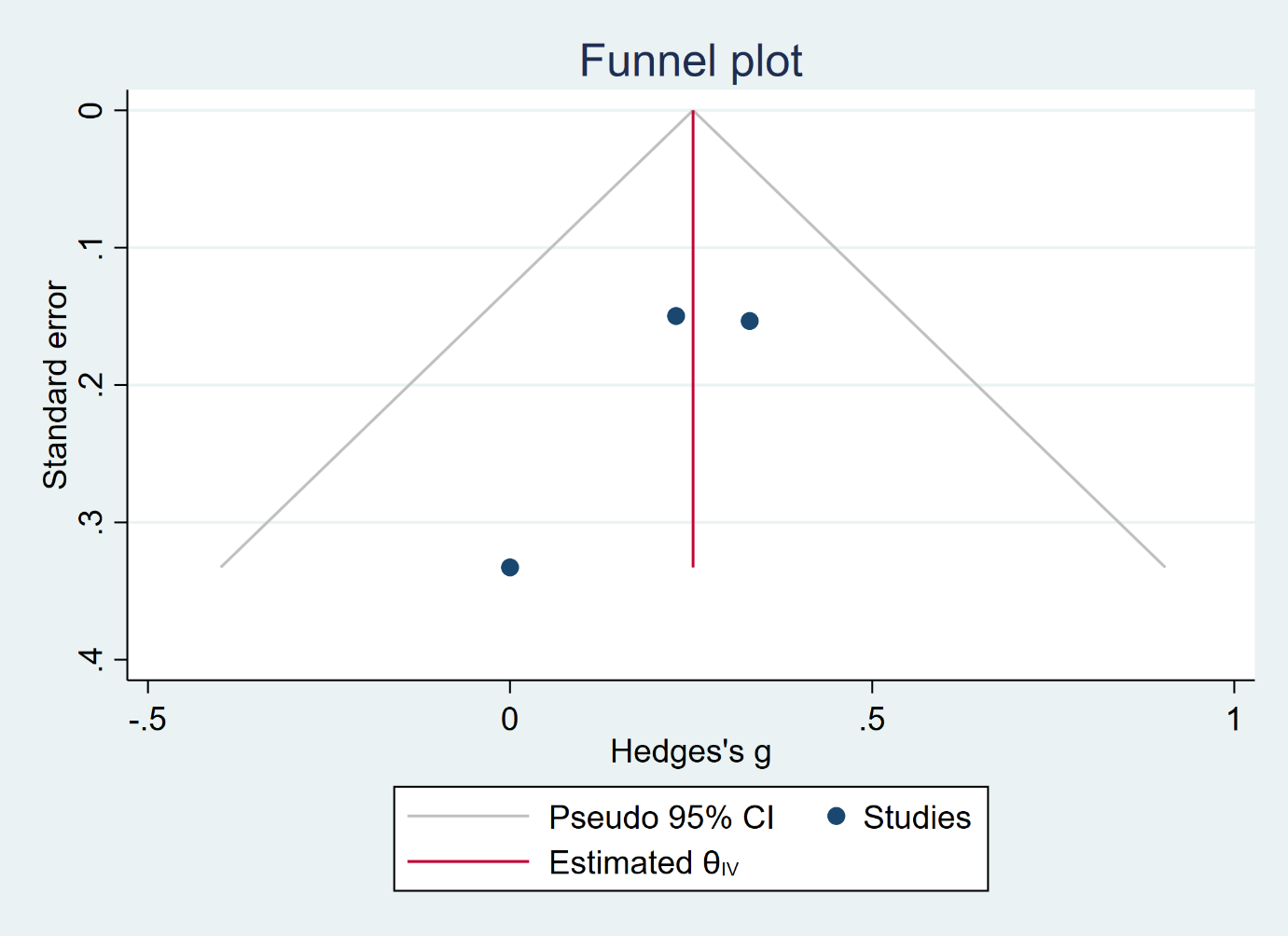


Figure S7. Funnel plot of the effects of exercise interventions on the overall domain of HRQoL compared with control conditions.


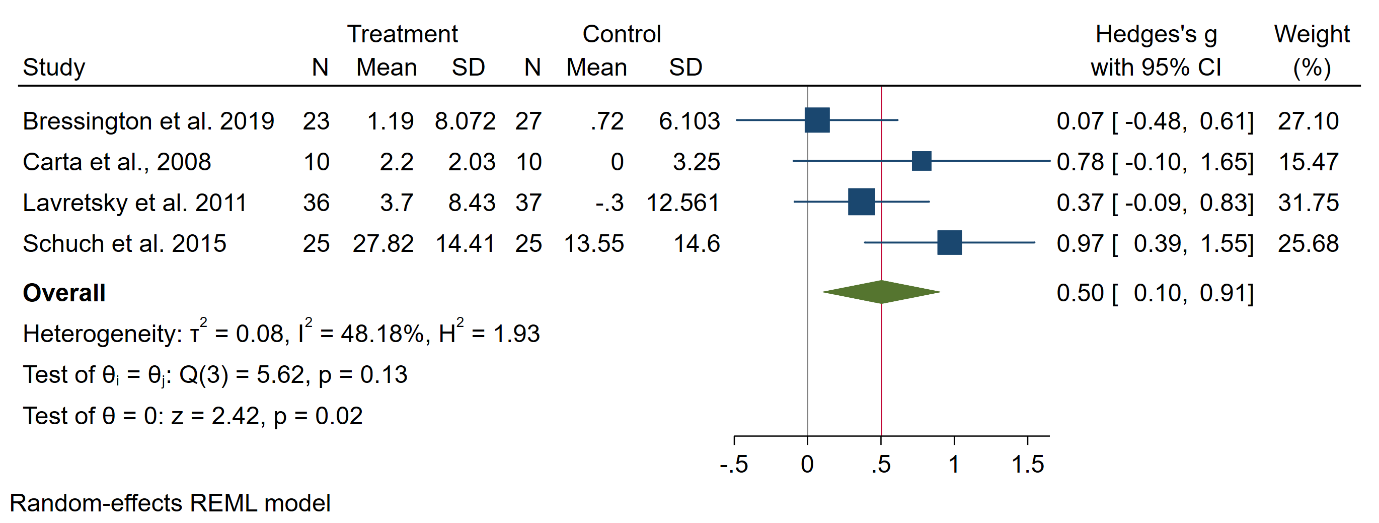


Figure S8. Meta-analysis of the effects of exercise interventions on the physical domain of HRQoL compared with control conditions.


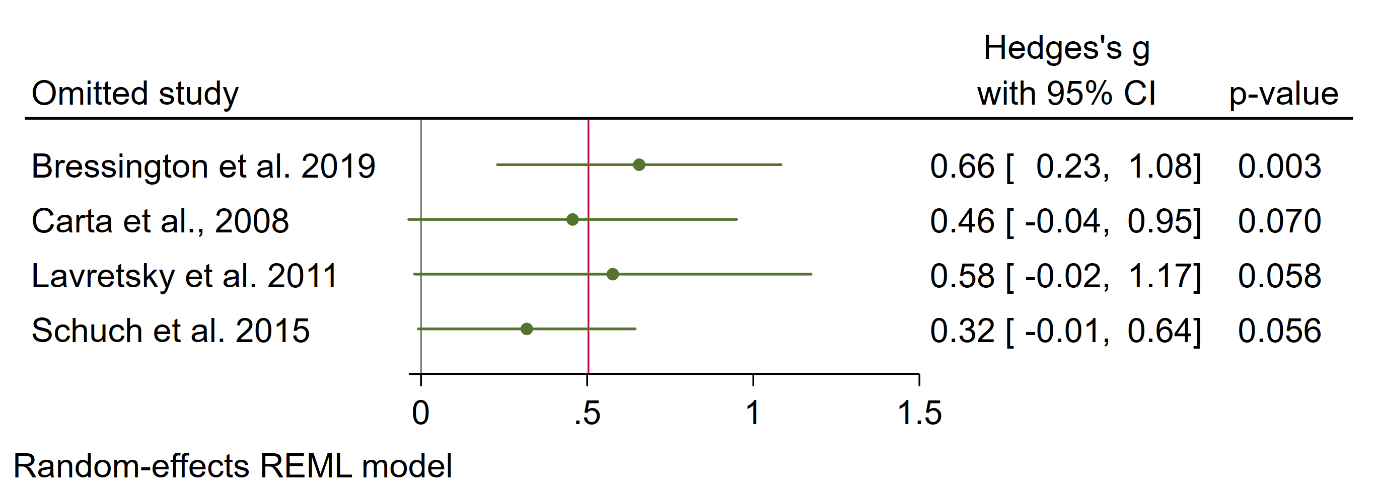


Figure S9. Sensitivity analysis of the effects of exercise interventions on the physical domain of HRQoL compared with control conditions.


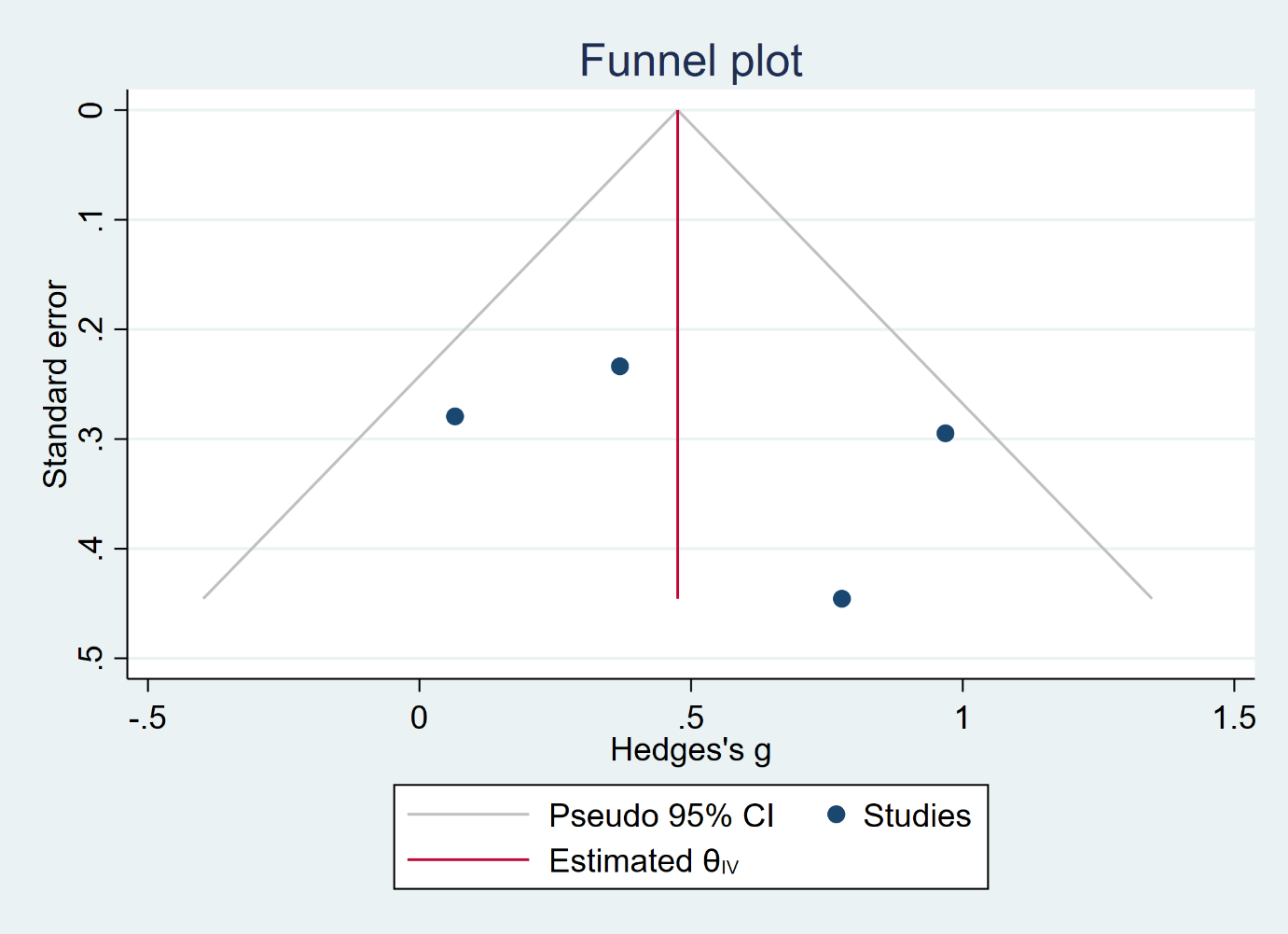


Figure S10. Funnel plot of the effects of exercise interventions on the physical domain of HRQoL compared with control conditions.


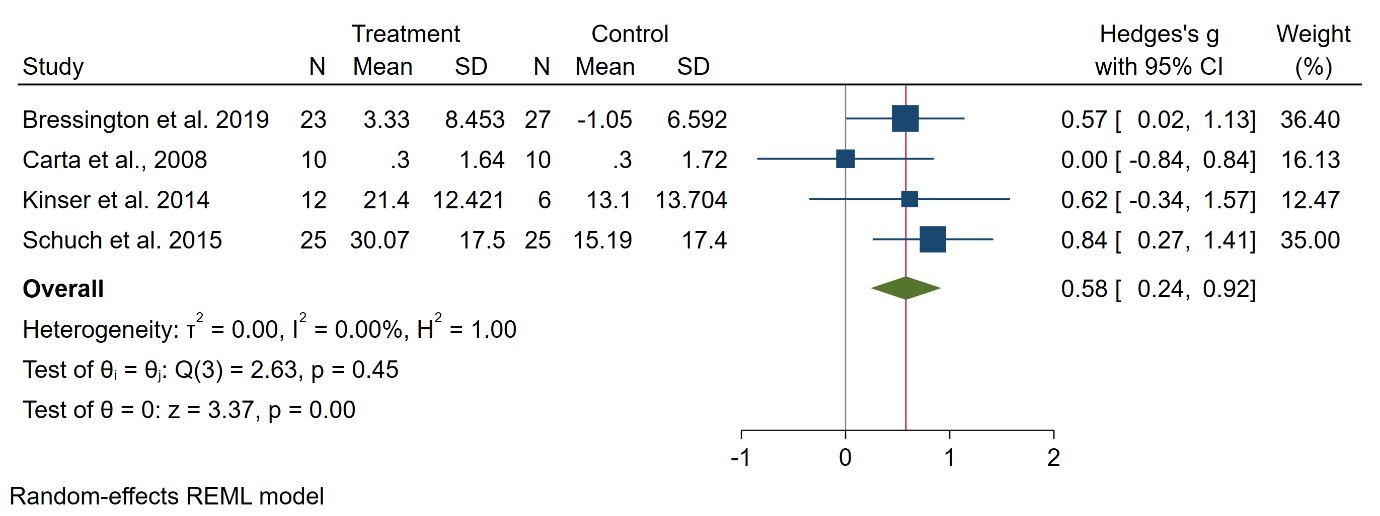


Figure S11. Meta-analysis of the effects of exercise interventions on the psychological domain of HRQoL compared with control conditions.


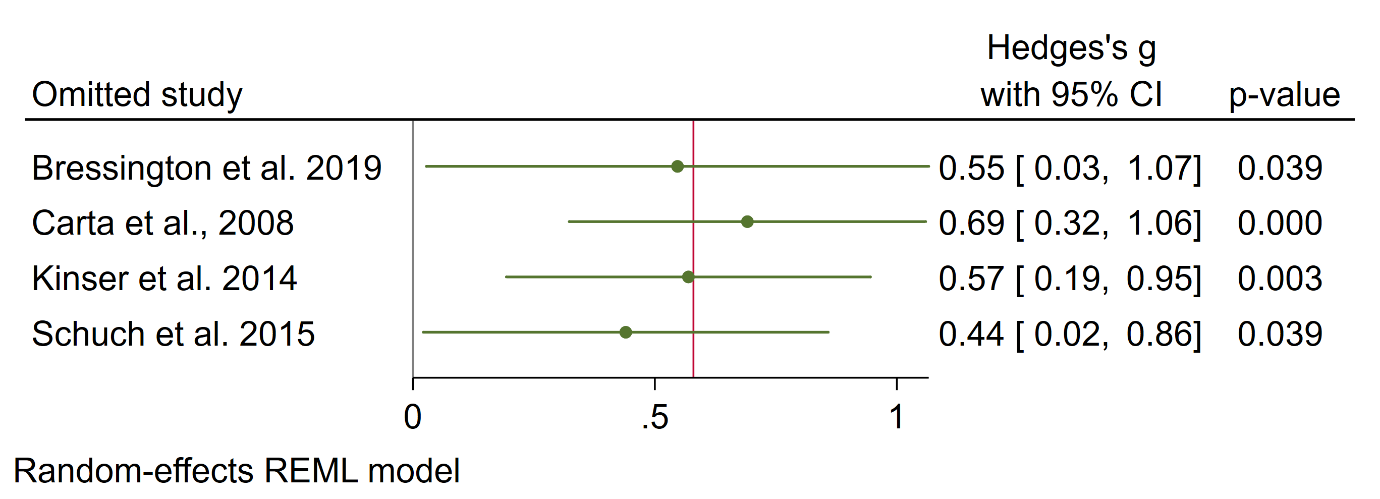


Figure S12. Sensitivity analysis of the effects of exercise interventions on the psychological domain of HRQoL compared with control conditions.


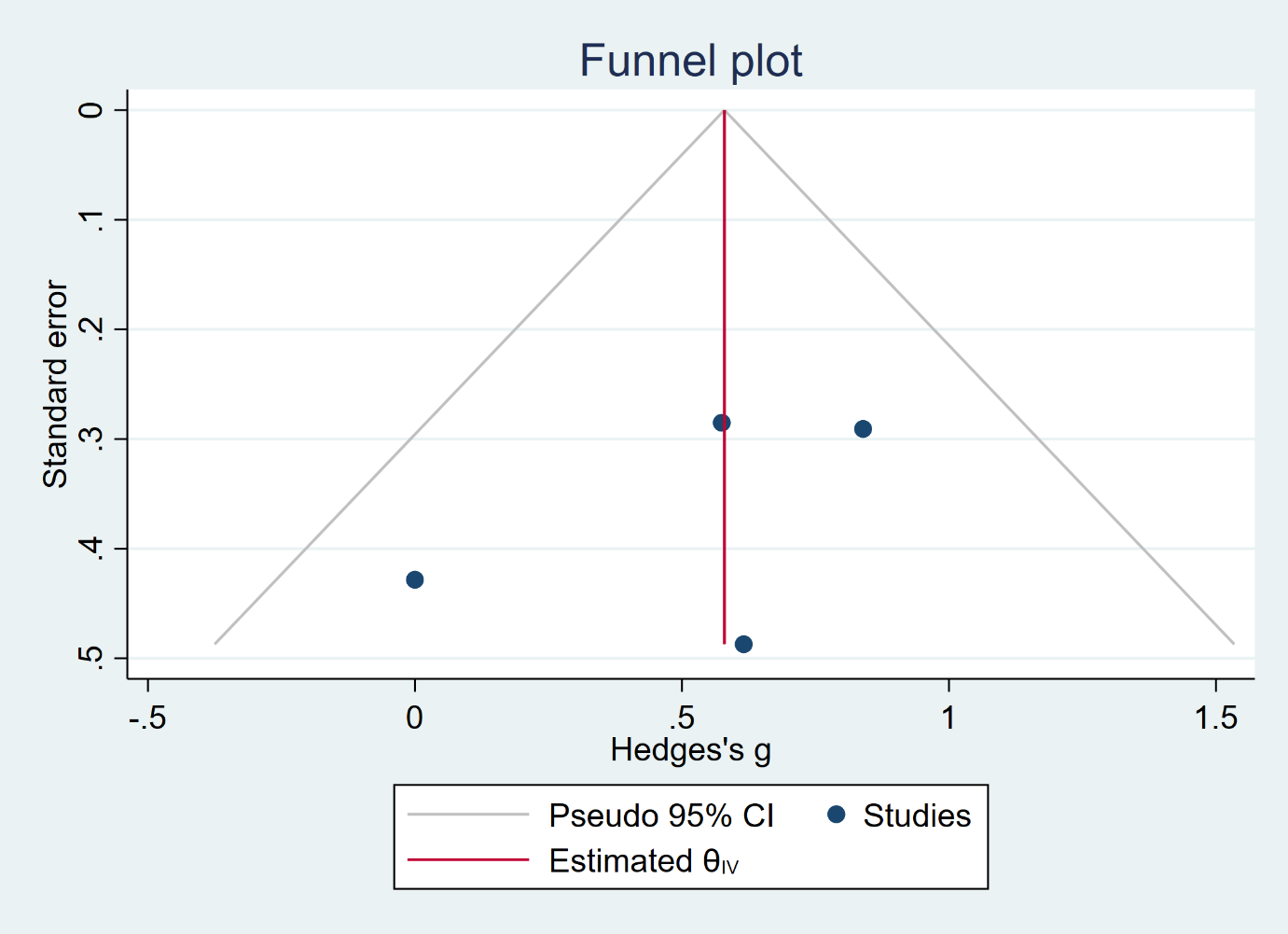


Figure S13. Funnel plot of the effects of exercise interventions on the psychological domain of HRQoL compared with control conditions.


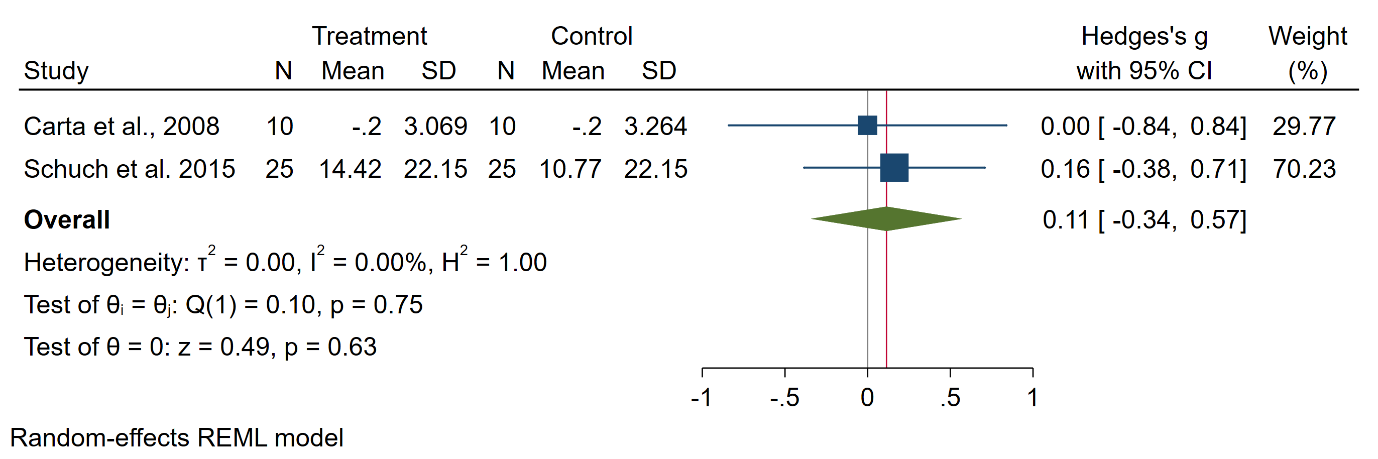


Figure S14. Meta-analysis of the effects of exercise interventions on the social domain of HRQoL compared with control conditions.


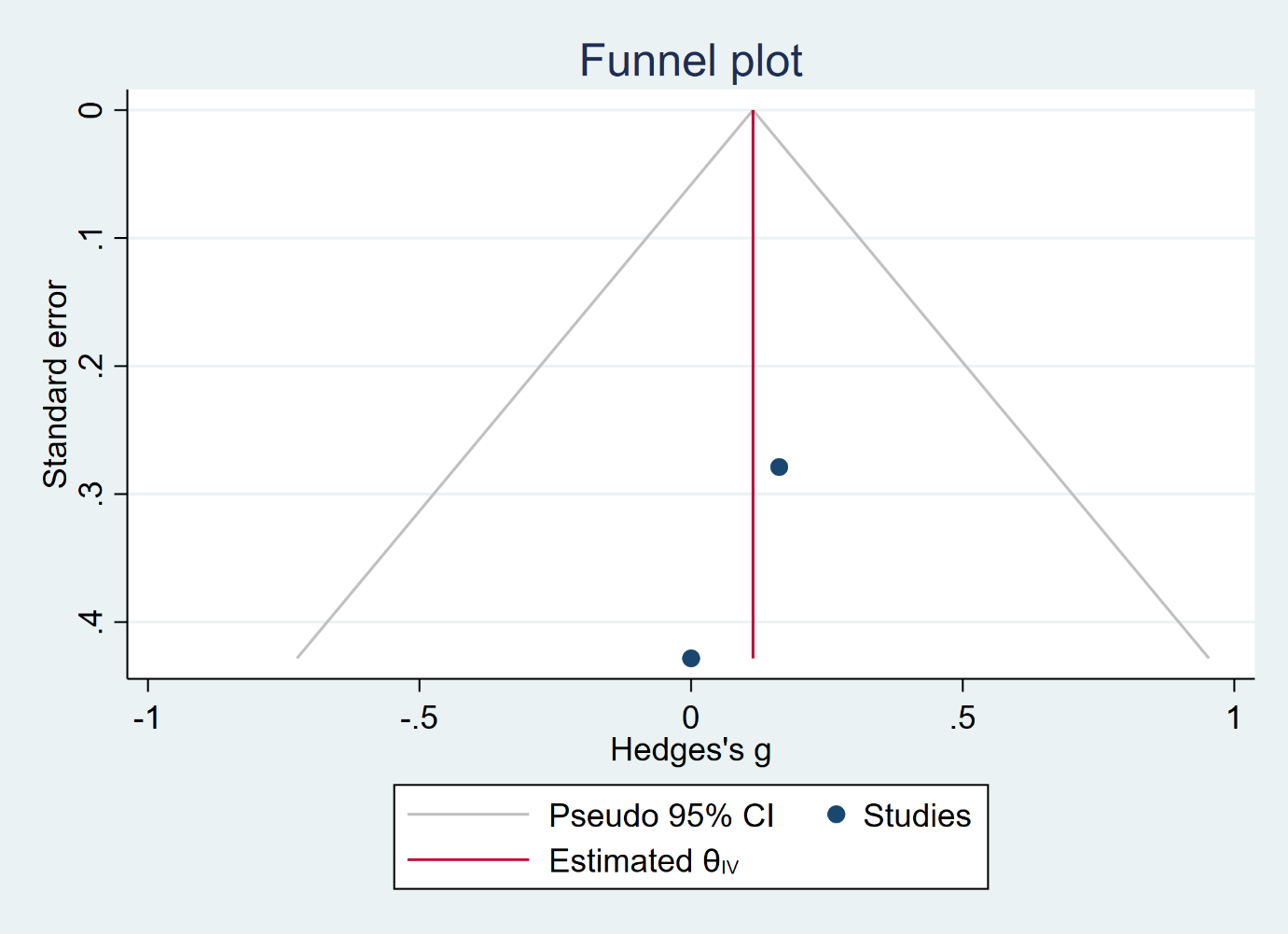


Figure S15. Funnel plot of the effects of exercise interventions on the social domain of HRQoL compared with control conditions.


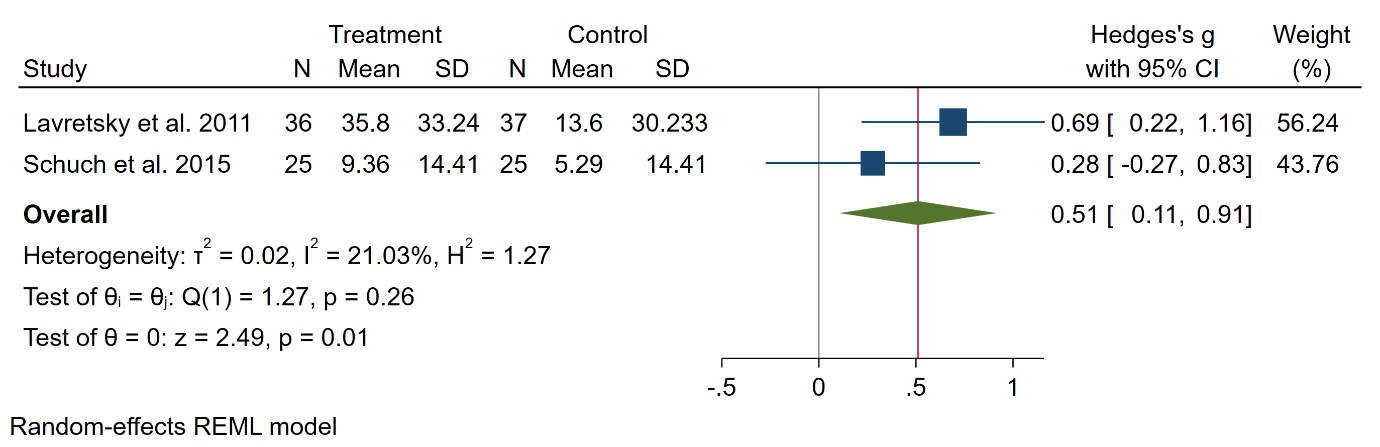


Figure S16. Meta-analysis of the effects of exercise interventions on the emotional domain of HRQoL compared with control conditions.


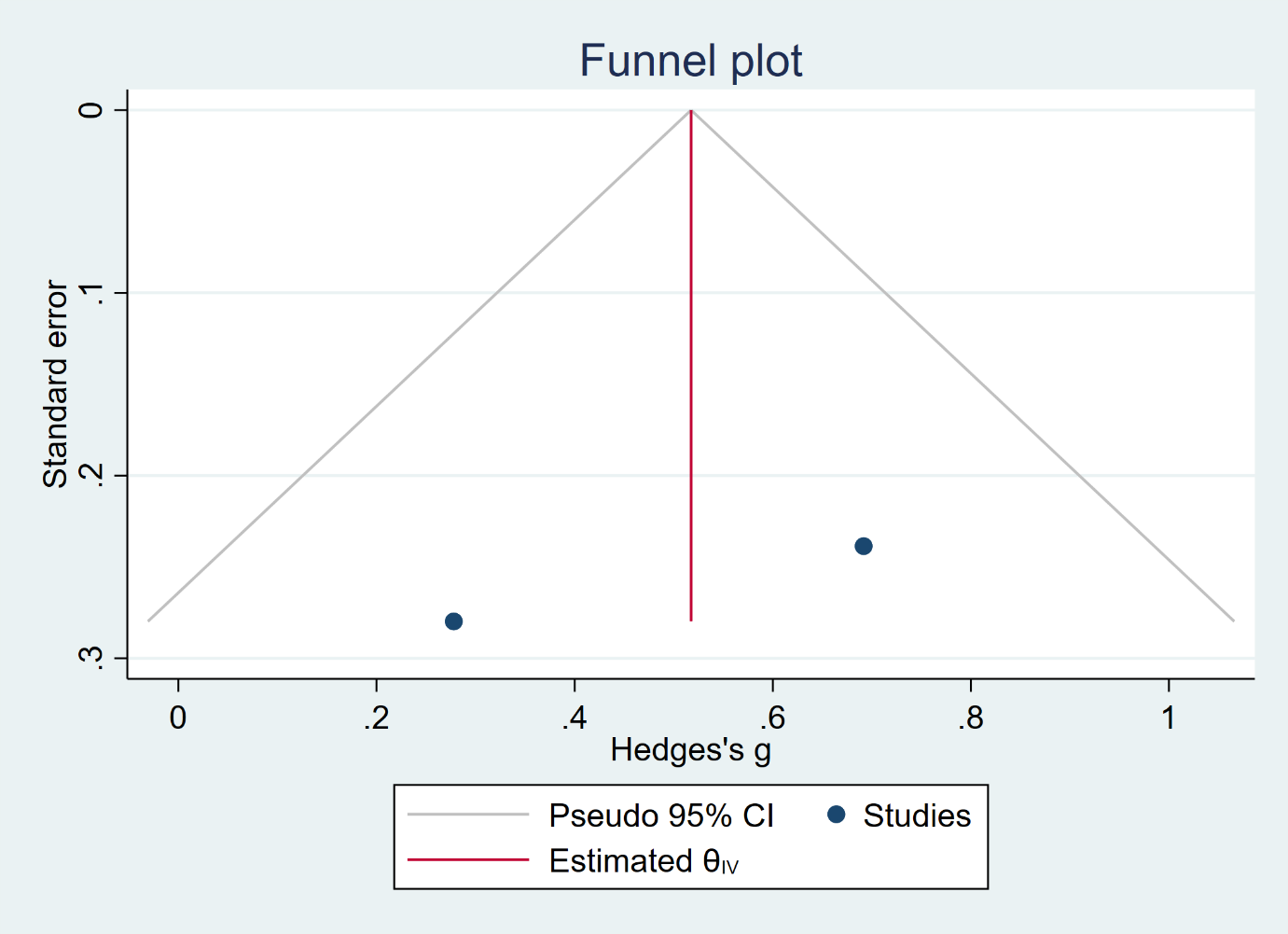


Figure S17. Funnel plot of the effects of exercise interventions on the emotional domain of HRQoL compared with control conditions.
